# Improving environmental monitoring of Vibrionaceae in coastal ecosystems through 16S rRNA gene amplicon sequencing

**DOI:** 10.1007/s11356-022-22752-z

**Published:** 2022-09-02

**Authors:** Elisa Banchi, Vincenzo Manna, Viviana Fonti, Cinzia Fabbro, Mauro Celussi

**Affiliations:** National Institute of Oceanography and Applied Geophysics - OGS, Via A. Piccard, 54, 34151 Trieste, Italy

**Keywords:** DNA, Metabarcoding, High-throughput sequencing, Amplicon sequence variant, EPA-ng, Gulf of Trieste, Pollution

## Abstract

The Vibrionaceae family groups genetically and metabolically diverse bacteria thriving in all marine environments. Despite often representing a minor fraction of bacterial assemblages, members of this family can exploit a wide variety of nutritional sources, which makes them important players in biogeochemical dynamics. Furthermore, several Vibrionaceae species are well-known pathogens, posing a threat to human and animal health. Here, we applied the phylogenetic placement coupled with a *consensus-based* approach using 16S rRNA gene amplicon sequencing, aiming to reach a reliable and fine-level Vibrionaceae characterization and identify the dynamics of blooming, ecologically important, and potentially pathogenic species in different sites of the northern Adriatic Sea. Water samples were collected monthly at a Long-Term Ecological Research network site from 2018 to 2021, and in spring and summer of 2019 and 2020 at two sites affected by depurated sewage discharge. The 41 identified Vibrionaceae species represented generally below 1% of the sampled communities; blooms (up to ~ 11%) mainly formed by *Vibrio chagasii* and *Vibrio owensii* occurred in summer, linked to increasing temperature and particulate matter concentration. Pathogenic species such as *Vibrio anguilllarum*, *Vibrio tapetis*, and *Photobacterium damselae* were found in low abundance. Depuration plant samples were characterized by a lower abundance and diversity of Vibrionaceae species compared to seawater, highlighting that Vibrionaceae dynamics at sea are unlikely to be related to wastewater inputs. Our work represents a further step to improve the molecular approach based on short reads, toward a shared, updated, and curated phylogeny of the Vibrionaceae family.

**Supplementary Information:**

The online version contains supplementary material available at 10.1007/s11356-022-22752-z.

## Introduction

Bacteria belonging to the Vibrionaceae family (Gammaproteobacteria) are present in all marine environments, with few species also found in brackish and freshwater ones (Thompson et al. 2004; Thompson and Polz 2006). They represent one of the best-studied models for the ecology and evolution of bacterial populations, also due to their easy culturability (Gomez-Gil et al. 2014; Takemura et al. 2014), and are an important component of marine ecosystems, contributing to the main biogeochemical cycles and supporting food webs (Chen et al. 2020).

Vibrionaceae are genetically and metabolically diverse, showing a wide range of lifestyles and niche specializations. Representatives of this family range from planktonic free-living organisms, to symbionts, to sessile forms attached to abiotic and biotic marine surfaces (e.g., suspended or sinking particles) including biofilm-forming species (Reen et al. 2006; Gomez-Gil et al. 2014; Palit and Nair 2013).

Vibrionaceae commonly represent a low fraction of bacterial assemblages in seawater. However, in response to different environmental factors (Takemura et al. 2014), they can reach very high densities forming transient (days) or long-lasting (weeks) blooms (Fuhrman et al. 2015; Westrich et al. 2016). Furthermore, several members of this family are recognized as human and animal pathogens (Thompson et al. 2004). Different Vibrionaceae species are associated with human intestinal and extraintestinal infections (Gomez-Gil et al. 2014; Monsreal et al. 2021) linked to the water microbiota and/or seafood; the most common pathogenic species are *Vibrio cholerae*, *Vibrio parahaemolyticus*, *Vibrio vulnificus*, and *Vibrio alginolyticus* (Baker-Austin et al. 2018). Other species such as *Vibrio harveyi*, *Vibrio anguillarum*, and *Vibrio splendidus* are commonly correlated with disease events in marine organisms (Austin and Zhang 2006; Rubio-Portillo et al. 2014), including commercially (e.g., aquaculture) and environmentally important fish, mollusc, and crustacean species (Frans et al. 2011; Vezzulli et al. 2015). Furthermore, Vibrionaceae represent the most abundant group in marine fish gut (Egerton et al. 2018) not only as pathogens, but also as symbionts in light organs, where they can exhibit bioluminescence (Urbanczyk et al. 2008; Burtseva et al. 2021).

Given the importance of this bacterial family, there is a high interest in studying its environmental distribution, dynamics, and public health implications (Stewart et al. 2008). In this perspective, culture-independent approaches such as amplicon sequencing (i.e., DNA metabarcoding), which allow to describe the biological diversity as a whole (Deiner et al. 2017), can be a valuable resource.

Amplicon sequencing uses high-throughput sequencing (HTS) to target a species-specific barcode region (Taberlet et al. 2012). The most common HTS platforms, such as Illumina and Thermo Fisher, can reach a read length of a few hundred (~ 600) nucleotides, not enough to cover the full length of the standard barcode regions (e.g., 16S rRNA, ITS) and therefore lowering their informative power. In fact, amplicon sequencing-derived data are generally assigned, when compared with taxonomy reference databases, up to the family or the genus level (Earl et al. 2018).

This limitation has brought to the development of tools that can increase the taxonomic resolution of HTS short reads (Matsen et al. 2012; Gwak and Rho 2020). Among these, the phylogenetic placement is one of the most interesting and increasingly used approach (Rajter and Dunthorn 2021). It has been successfully applied to place the short reads on phylogenetic trees built using full-length reference sequences, allowing their assignment at species level (Matsen et al. 2012; Janssen et al. 2018), showing a greater accuracy than de novo (i.e., based only on short reads) tree construction (Balaban et al. 2020).

Different biomonitoring studies in aquatic environments have used this approach for the survey of metazoans (Mitsi et al. 2019), protists (Elferink et al. 2017; Keck et al. 2018; Gottschling et al. 2021), and prokaryotes (López-Cortés et al. 2020; Iniesto et al. 2021). Recently, a comprehensive and curated cyanobacterial reference database that can be readily used for the phylogenetic placement of these organisms was released (*Cydrasil*; Roush et al. 2021), widening the biomonitoring potential of this tool.

Here, we present the application of the phylogenetic placement coupled with a *consensus-based* approach intending to reach a reliable and fine (i.e., at species level) taxonomic characterization within the family Vibrionaceae in seawater and depuration plants at different sites of the northern Adriatic Sea using amplicon sequencing of the 16S rRNA gene. The aim of this work is to characterize their composition and relative abundance to identify the dynamics of blooming, ecologically important, and potentially pathogenic species.

## Materials and methods

### Study area and sampling

**Fig. 1:**
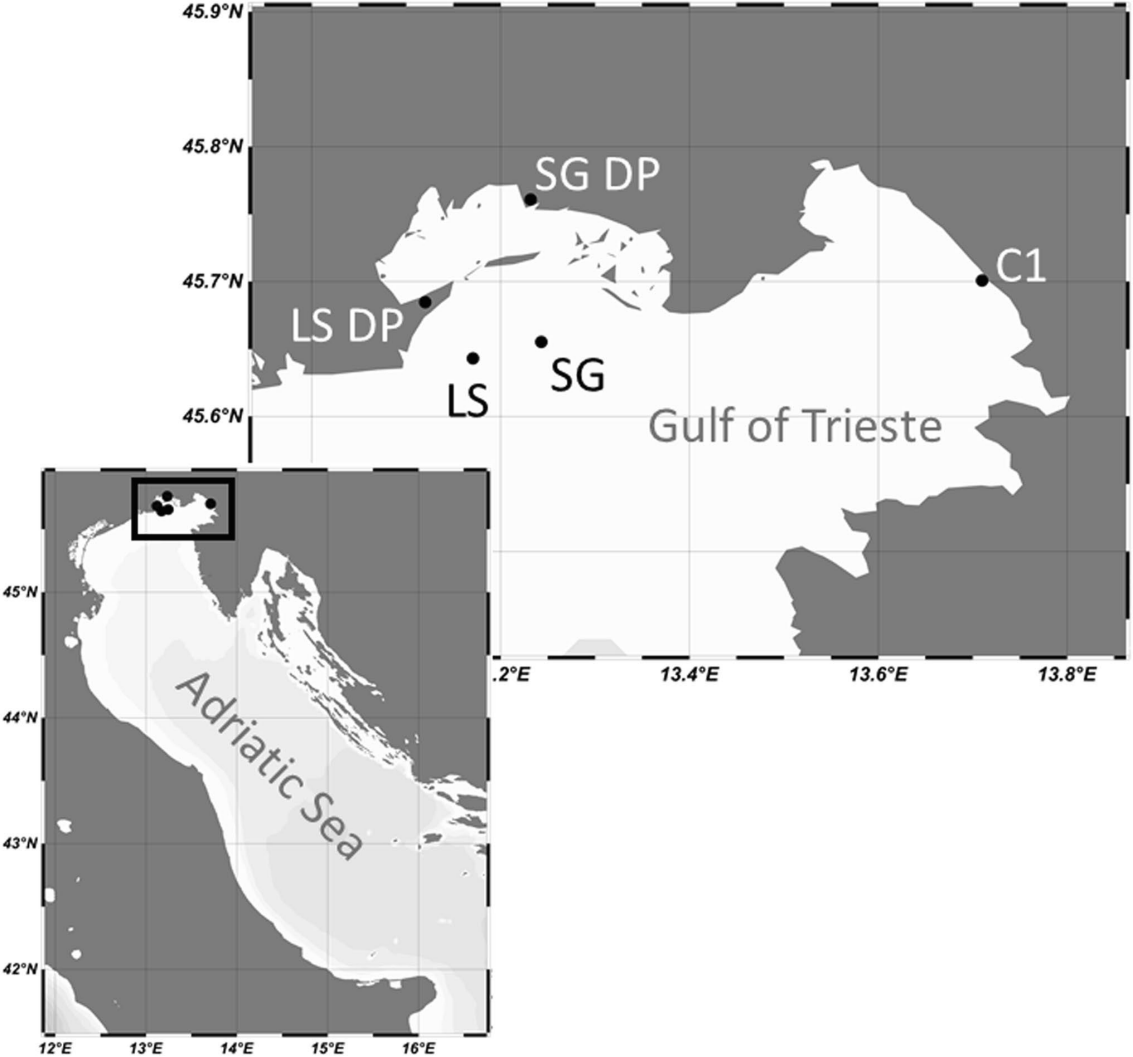
Sampling sites in the North Adriatic. LS, Lignano Sabbiadoro; SG, San Giorgio di Nogaro; DP, depuration plant

In this work, we studied three coastal marine sites in the northern Adriatic Sea (Fig. 1; Schlitzer 2018).

The C1 monitoring station, under study within the Long-Term Ecological Research network (LTER), is located in the Gulf of Trieste (C1, 45° 42′ 2″ N, 13° 42′ 36″ E). Samples investigated at this station were collected monthly between October 2018 and July 2021, at ~ 1 m (surface, S) and ~ 15 m (at the bottom of the water column, B) depths, using 5-L Niskin bottles.

The other two sampling sites (Lignano Sabbiadoro, LS, 45° 38′ 35″ N, 13° 10′ 14.5″ E and San Giorgio di Nogaro, SG, 45° 39′ 19″ N, 13° 14′ 39″ E) are directly affected by the discharge of depurated sewage from the wastewater treatment plants (WWTP) located in their respective municipalities. Both WWTP include primary and secondary treatment of urban and industrial wastewater and a final disinfection step (with UV plus peracetic acid in LS WWTP, with only peracetic acid in SG WWTP). Their marine outfall pipes discharge at 7.5 and 10 km from the shoreline, at 13.7 and 14.3 m depth, respectively. Both sites were sampled monthly in spring and summer 2019 and 2020 (i.e., from April to September/October), using 5-L Niskin bottles. In both cases, seawater samples were collected close to the WWTP submarine outfall pipelines, 1 m above the main diffusion point.

In addition to seawater samples, in LS and SG, we analyzed samples of treated sewage collected upstream the injection into the discharging pipeline of depurators.

Seawater and depuration plant (DP) samples were filtered through 0.2-μm PES membrane filters (PALL Laboratory) and stored at − 80 °C until further processing. Filtration was performed until clogging of membrane pores. Filtered volumes were in the range of 1–3 L for seawater (SW) and 70–500 mL for DP.

### Ancillary parameters

At C1 station, a set of biogeochemical parameters, including water temperature, salinity, chlorophyll *a* (Chl *a*) and particulate organic carbon (POC) concentration, and prokaryotic abundance were determined contextually to DNA sampling (see the “Study area and sampling” section).

Temperature and salinity were measured by means of a multiparametric probe (SBE 19plus SEACAT), calibrated every 6–12 months.

Chl *a* concentration was determined fluorometrically according to Lorenzen and Jeffrey (1980) as detailed in Manna et al. (2021). POC concentration was measured using an elemental Analyser CHNO-S Costech mod. ECS 4010 applying the methods by Pella and Colombo (1973) and Sharp (1974), as detailed in Celussi et al. (2017).

Total prokaryotes were counted by flow cytometry, using a FACSCanto II (Becton Dickinson) instrument, equipped with an air-cooled laser at 488 nm and standard filter setup. The method by Marie et al (1999) was used, as detailed in Manna et al. (2021).

### DNA extraction, 16S amplicon sequencing, and taxonomic assignment

DNA was extracted from membrane filters using the DNeasy PowerWater Kit (Qiagen) with some modifications to increase the DNA yield and quality (detailed in Celussi et al. 2018). Extracted DNA was quantified with a Qubit Fluorimeter (Thermo Fisher Scientific).

For the amplicon sequencing, the V4–V5 region of 16S rRNA gene was amplified using 515-Y (5′-GTGYCAGCMGCCGCGGTAA-3′) and 926R (5′-CCGYCAATTYMTTTRAGTTT-3′) primers (Parada et al. 2016) provided with Illumina adaptors. Libraries were prepared following the 16S Metagenomic Sequencing Library Preparation protocol (Amplicon 2013) and run on an Illumina MiSeq System for a read length of 2 × 250 bp at the genetic and epigenetic ARGO Open Lab Platform, Area Science Park, Trieste, Italy.

Raw sequences were quality filtered and denoised with DADA2 v. 1.20.0 (Callahan et al. 2016) in R (v. 4.4.1; R Core Team 2021) with pseudo-pooling method⁠. After primer removal and visual inspection of the read quality profiles, forward and reverse reads were truncated at positions 220 and 190, respectively. Chimeric sequences were identified and removed in consensus mode. Amplicon-sequence variants (ASVs) with frequency < 2 (singletons) were removed.

Taxonomy was assigned using the Sklearn Naïve Bayes taxonomy classifier (Bokulich et al. 2018) against the SILVA 99% reference database with 7-level taxonomy release 138 (Quast et al. 2012) in QIIME2 2020.6 (Bolyen et al. 2019). We identified 132 oligotypes (ASVs) belonging to Vibrionaceae family: 57 in C1 surface (C1_S), 83 in C1 bottom (C1_B), 66 in Lignano Sabbiadoro (LS), 62 in San Giorgio di Nogaro (SG), 14 in Lignano Sabbiadoro depurator plant (LS_DP), and 9 in San Giorgio di Nogaro depurator plant (SG_DP).

Vibrionaceae oligotypes identified with SILVA were further taxonomically assigned with other reference databases: GTDB ssu_r86.1_20180911 (https://osf.io/25djp/wiki/home/) using the same approach described above in QIIME2, with SILVA release 138 (Quast et al. 2012) with last common ancestor method (LCA), and Ribosomal Database Project (RDP; Cole et al. 2007) using SINA v1.2.11 at default parameters (Pruesse et al. 2012). BLASTN 2.12.0 + (Altschul et al. 1997) was also used, aligning the oligotypes against the nucleotide collection and excluding uncultured and environmental sample sequences: the hits with the lower e-value, 100% query cover, and > 97% of identity were selected.

### Consensus-based workflow

To maximize the reliability of the species-level assignment, we applied a *consensus-based* approach (Fig. S1). Firstly, the Vibrionaceae oligotypes were used for the phylogenetic placement (see the following section). Then, an oligotype selection was performed, retaining only the ones in which the phylogenetic placement at the genus level agreed with at least 3 out of the 5 reference databases, while the others were considered “unassigned” Vibrionaceae. In this way, we exploited the power of the phylogenetic placement to reach the lower taxonomic level, while acknowledging the limits of short read sequencing.

### Phylogenetic placement and phylogenetic tree construction

The phylogenetic placement was used to assign the Vibrionaceae oligotype sequences to the near full-length 16S rRNA gene reference phylogeny. The input to the phylogenetic placement algorithm consists of a reference tree, a corresponding reference multiple sequence alignment, and a collection of query sequences. The output is a set of assignments of the query sequences to branches of the tree (Matsen et al. 2012).

For the reference tree, Vibrionaceae sequences were retrieved from Gomez-Gil et al. (2014) and integrated with other lineages and species more recently characterized. The final dataset (Table S1) included 173 sequences coming from published, curated, and verified data, 169 of which were type strains. Reference sequences were aligned with mafft v. 7 and G‐INS‐I strategy (Katoh and Standley 2013). The resulting alignment was used for tree construction. A maximum likelihood (ML; Felsenstein 1981) tree was inferred using RAxML v.8.2.10 (Stamatakis 2014) with the parameters suggested for the evolutionary placement algorithm (EPA-ng; Barbera et al. 2019; Czech et al. 2022). The general time-reversible model and the discrete gamma distribution of nucleotide substitution frequencies (GTR + GAMMA) model of nucleotide substitution were used; supporting values were estimated from 500 bootstraps (Felsenstein 1985). The query sequences were the 132 Vibrionaceae oligotypes. To verify the reliability of the phylogenetic placement and the influence of query length, sequences belonging to three *Vibrio* species isolated in the Gulf of Trieste (Fabbro et al. 2010, 2012), *V. anguillarum* (accession number GU120676.1, 843 bp), *V. chagasii* (GU120679.1, 923 bp), and *V. parahaemolyticus* (GQ332281.1, 363 bp) were added as controls. The *V. anguillarum* and *V. chagasii* sequences were included in the query list both in their original length and in a trimmed version comparable to the sequences coming from NGS (~ 374 bp, named “short”) after aligning them with MUSCLE at default parameters (Edgar 2004) in MEGA X (Kumar et al. 2018).

The query and the control sequences were aligned against the reference alignment using the PArsimony-based Phylogeny-Aware Read Alignment program (PaPaRa) v. 2.5 (Berger and Stamatakis 2012). Then, EPA-ng (Barbera et al. 2019) was used to split query and reference from the PaPaRa alignment. The best scoring RAxML reference tree was selected as input for the phylogenetic placement of the query sequences. For each oligotypes, the placement with the higher likelihood was selected, using a conservative consensus approach following Hoffmann et al. (2020). The “jplace” file (Berger and Stamatakis 2011) was visualized and edited with Interactive Tree Of Life (iTOL) v5 (Letunic and Bork 2021).

To further explore the relationship among Vibrionaceae oligotypes and check their similarity with control sequences, a phylogenetic tree was constructed with mafft v. 7 and G‐INS‐I strategy (Katoh and Standley 2013) and inferring a maximum likelihood (ML) tree using RAxML v.8.2.10 (Stamatakis 2014), with the GTR + GAMMA model of nucleotide substitution.

### Statistical analyses

To investigate potential environmental drivers of Vibrionaceae distribution, we analyzed the correlation patterns between the absolute abundance of Vibrionaceae ASVs and temperature, salinity, Chl *a*, and POC concentration. Absolute abundance was calculated by multiplying the total prokaryotic abundance, measured by flow cytometry (see the “Ancillary parameters” section), by the relative abundance of Vibrionaceae ASVs in the LTER-C1 dataset (Mena et al. 2021).

Since data revealed to be not normally distributed (Shapiro–Wilk test, *p* < 0.05), we used Spearman’s correlation metrics. A *p*-value ≤ 0.05 was considered significant, under the null hypothesis that ranks of absolute Vibrionaceae abundance did not covary with the ranks of the environmental parameters considered.

Data handling, visualization, and analysis were conducted in the R environment (v. 4.4.1; R Core Team 2021) using the packages *tidyverse* (Wickham et al. 2019) and *phyloseq* (McMurdie and Holmes 2013).

## Results

### Phylogenetic placement and Vibrionaceae species-level assignment

**Fig. 2:**
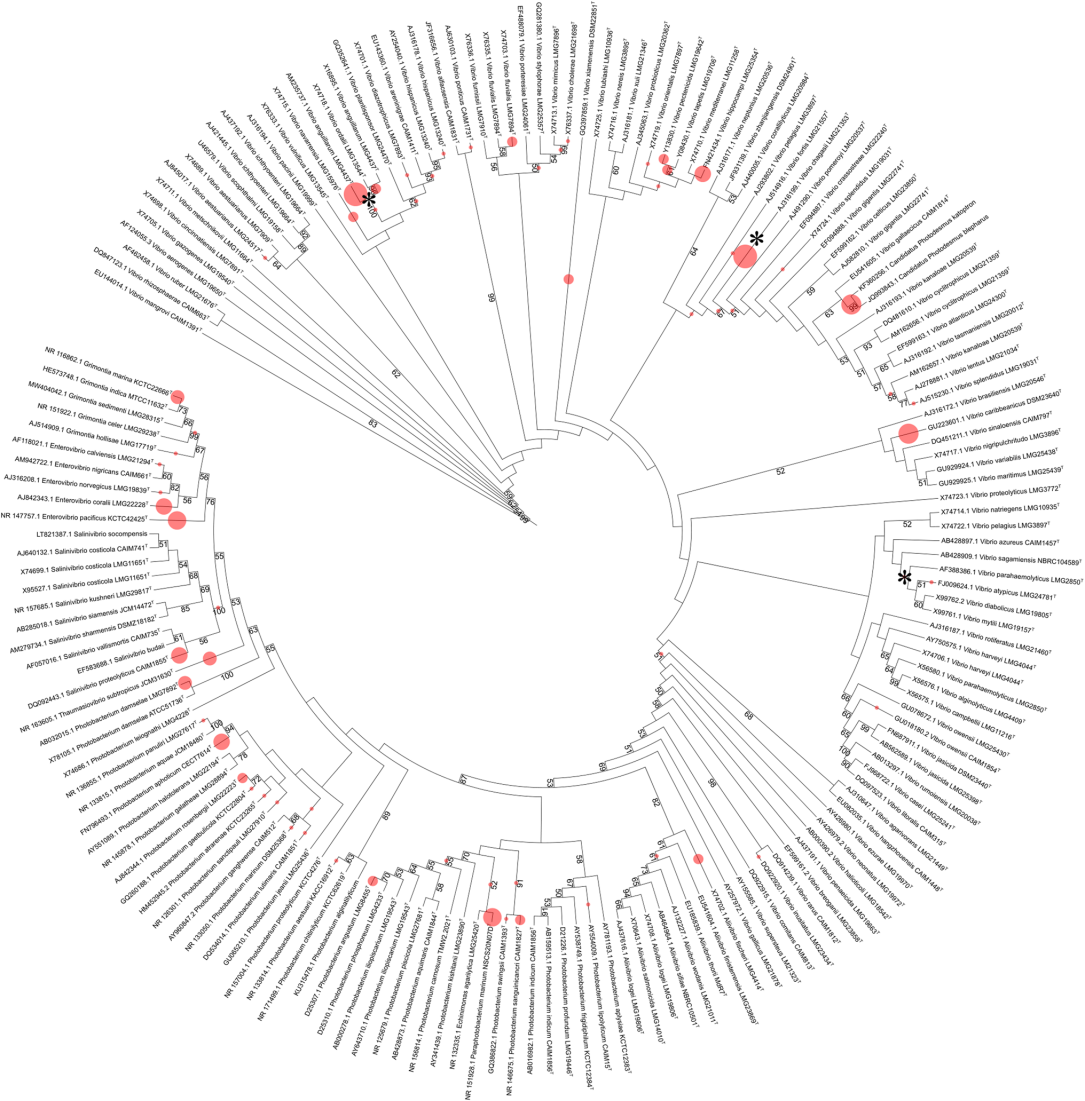
Phylogenetic placement of 132 Vibrionaceae oligotypes. The phylogeny was inferred from 173 reference sequences, using maximum likelihood under the GTR + GAMMA model. Support values ≥ 50% (bootstrap *n* = 500) are showed. Type strains are indicated by the index “T.” The radius of red circles is proportional to the number of oligotypes placed (min = 1, max = 12). Asterisks represent the placement of the known Vibrionaceae species used as controls

The phylogenetic tree was composed by 173 sequences with a length of 1770 bp (Fig. 2). Significant bootstrap values (≥ 70; Van de Peer and Salemi 2009) were concentrated in clades belonging to *Aliivibrio*, *Enterovibrio*, *Grimontia*, and *Salinivibrio*, whereas *Vibrio* clades were generally less supported. This has already been noted in the 16S rRNA gene-based phylogenies of the Vibrionaceae, which showed low divergence particularly in the genus *Vibrio* (Gabriel et al. 2014; Ashok Kumar et al. 2020).

The phylogenetic placement of the Vibrionaceae oligotypes was performed using the reference tree. The 132 Vibrionaceae oligotypes were placed in 9 genera and 39 species (Fig. 2); 97 oligotypes were assigned at the species level, 12 were assigned to more than one species of the same genus, while for 23 oligotypes, the placement was possible only up to the genus level (Table S2).

The placement of control sequences of known attribution allowed us to obtain a measure of the goodness of the workflow (Fig. 2). The *V. parahaemolyticus* sequence was correctly attributed to V. parahaemolyticus clade. Both the *Vibrio anguillarum* original and trimmed (“short”) sequences were attributed to *V. anguillarum/V. ordalii* clade. *Vibrio chagasii* original sequence was attributed correctly up to the species level, while the trimmed one (i.e., simulating the corresponding short reads in our dataset) was placed also in the upper node. This helped us to place 8 query sequences correctly in *V. chagasii* species.

Different levels of agreement were detected from the comparison of the taxonomic assignment at genus species between the phylogenetic placement and the 5 databases (Table S3).

Of the 132 oligotypes, 71 (54%) presented a total consensus. The phylogenetic placement assignments at the genus level agreed with BLAST assignment for 98 (74%) oligotypes, with SILVA LCA for 93 (70%), with SILVA for 89 (67%), with GTDB for 81 (61%), and with RDP for 78 (59%).

After this comparison, 103 (78%) oligotypes were retained as they presented a consensus between the phylogenetic placement in at least 3 out of 5 databases.

The oligotypes that were removed at this step belonged to 10 species (Table S4). The fraction of removed reads ranged from low (0–5%) for *Vibrio ippocampi*, *Photobacterium sanguinicancri*, and *V. anguillarum*, to medium (5–30%) for *P. aphoticum*, *Thaumasiovibrio subtropicus*, and *P. damselae*, and to high (30–90%) for *Enterovibrio pacificus*, *Salinivibrio proteolyticus*, and *Paraphotobacterium marinum* (this latter as the only species that was removed). Overall, they represented ~ 3% of the total Vibrionaceae reads.

Finally, we filtered out the oligotypes (18) not assigned at the species level; we reached a final dataset composed by 85 oligotypes (65% of the initial dataset, representing the ~ 75% of the total Vibrionaceae reads; Table S3).

The oligotypes’ phylogenetic tree (Fig. S2) was used to check whether the ASVs assigned to the same clades of the control sequences were correctly retained in the final dataset. No oligotype clustered with the *V. parahaemolyticus* control sequence in the phylogenetic tree and none was assigned to this species by the phylogenetic placement. For *V. anguillarum*, 10 out of 10 oligotypes retained (ASV_33,44,56,71,75,77,81,99,100,125) clustered together with this sequence in the phylogenetic tree. ASV_90 that was assigned to this species with the phylogenetic placement and discarded within the *consensus-based* approach was placed outside the clade. For *V. chagasii*, 7 out of 7 oligotypes retained (ASV_01,23,29,40,70,94,131) clustered together with this sequence in the phylogenetic tree. ASV_130, which was assigned to this species with the phylogenetic placement and discarded with our *consensus-based* approach, was placed outside the clade.

### Presence and distribution of Vibrionaceae species

The final dataset was composed by 85 oligotypes (out of the initial 132) corresponding to 47 species (including 6 clades): one species of *Aliivibrio*, four of *Enterovibrio*, one of *Grimontia*, one of *Salinivibrio*, one of *Thaumasiovibrio*, 14 of *Photobacterium*, and 25 of *Vibrio*. Overall, in C1_S and C1_B, 28 and 31 species were detected, respectively. In LS and SG, 36 and 33 species were detected. In LS_DP and SG_DP, 11 and 6 species were detected.

**Fig. 3:**
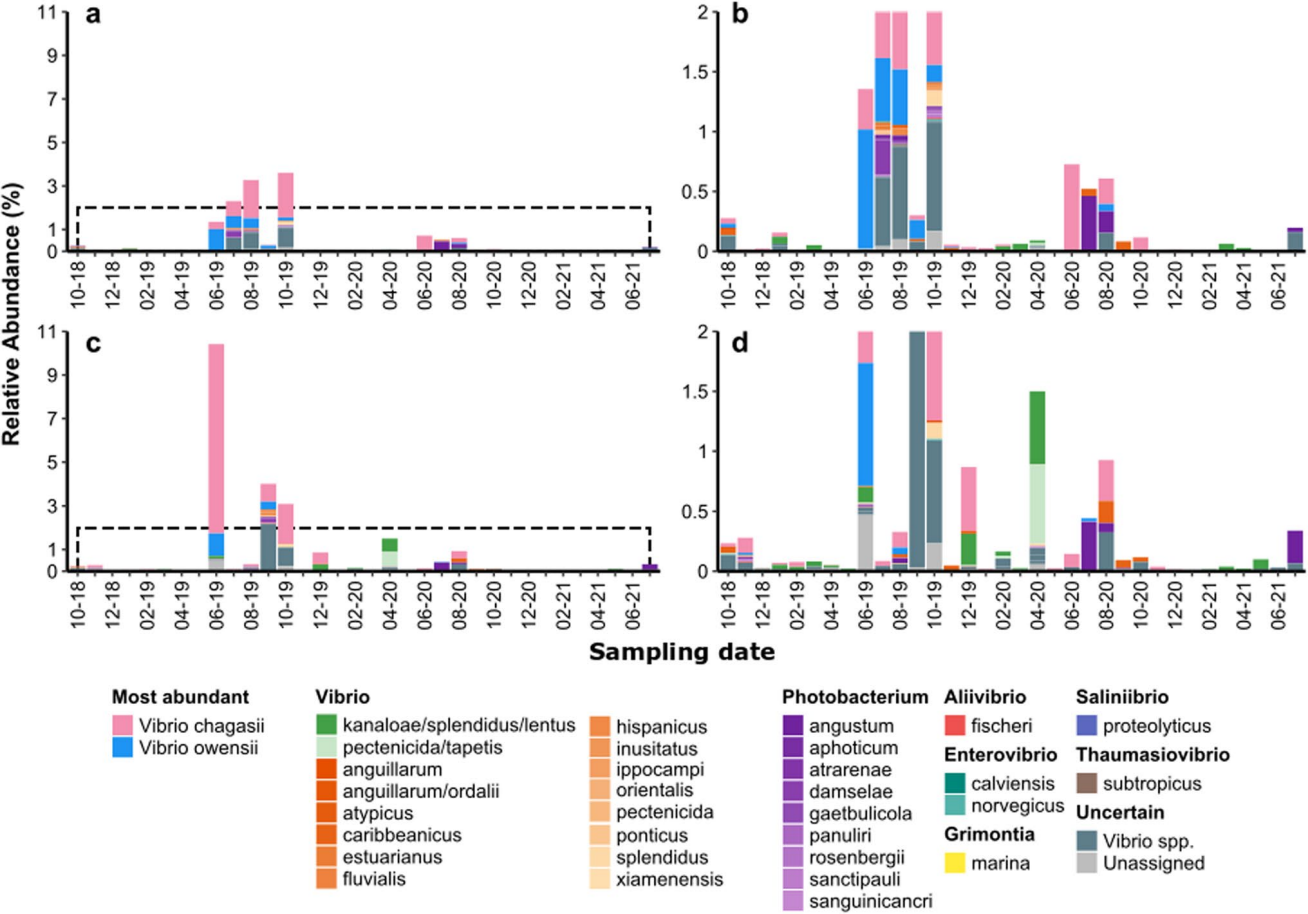
Relative abundance of Vibrionaceae species in seawater samples of C1 surface (**a** and **b**) and bottom (**c** and **d**). The panels **b** and **d** zoom on the abundances up to 2%, dashed in panels **a** and **b**, respectively

**Fig. 4:**
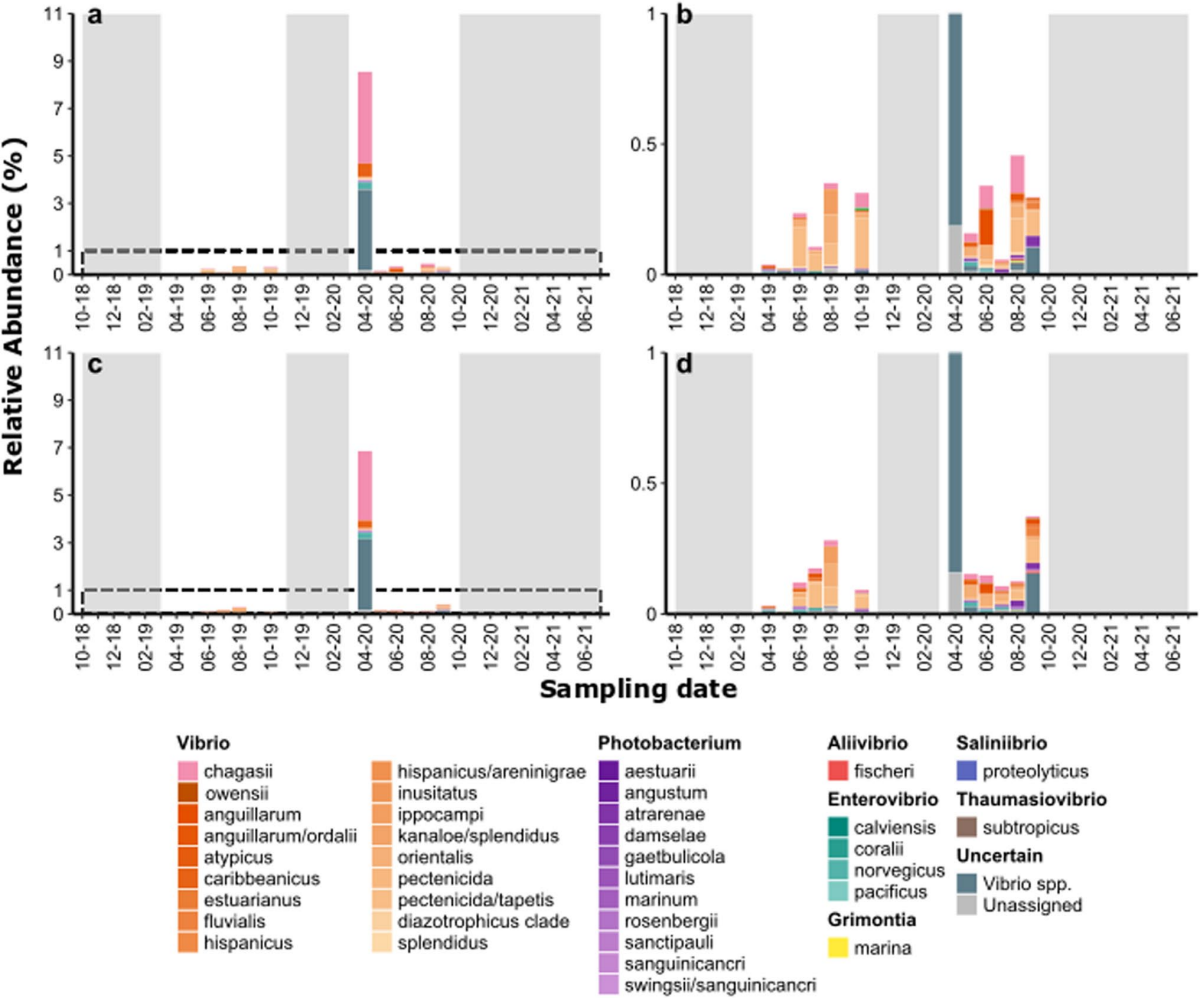
Relative abundance of Vibrionaceae species in seawater samples of Lignano Sabbiadoro (**a** and **b**) and San Giorgio di Nogaro (**c** and **d**). The panels **b** and **d** zoom on the abundances up to 1%, dashed in panels **a** and **b**, respectively. Gray areas indicate months that were not sampled

**Fig. 5:**
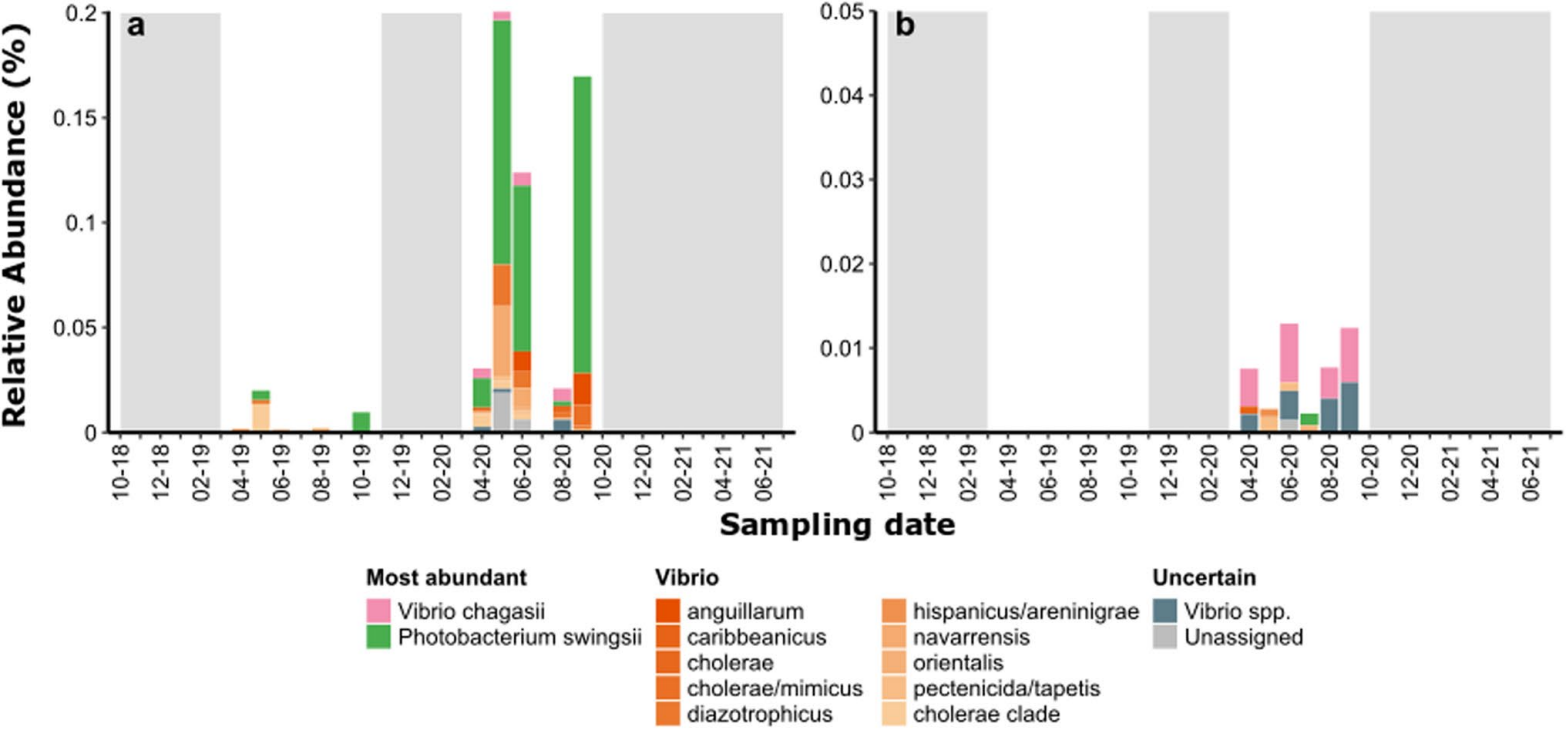
Relative abundance of Vibrionaceae species in depuration plant samples of Lignano Sabbiadoro (**a**) and San Giorgio di Nogaro (**b**). Gray areas indicate months that were not sampled

The taxonomic composition and relative abundance in each dataset are reported in Figs. 3, 4, and 5 and Table S5.

Regarding seawater samples, in C1 surface water (S), the average abundance of Vibrionaceae was 0.4 ± 0.9% with a peak of ~ 3% in June, July, August, and October 2019 (Fig. 3). In bottom water (B), the average abundance was 0.7 ± 1.9% with a peak of ~ 10% in June 2019 (Fig. 3).

In LS, the average abundance was 0.5 ± 0.6%, while in SG was 0.5 ± 0.6%. In seawater of both sites, a peak (~ 8%) was recoded in April 2020 (Fig. 4).

In all sites, the Vibrionaceae blooms were mainly composed by *Vibrio chagasii*, and, in minor proportion in C1 site, by *V. owensii* (Fig. 3 and 4).

The relative abundances of Vibrionaceae in the depurator samples were lower than in seawater samples by a few orders of magnitude, with no relevant peaks over time (Fig. 5). The average relative abundances were 9.7 ± 0.5 × 10^−4^% and 5.0 ± 0.3 × 10^−5^%, for LS_DP and SG_DP, respectively.

### Relationships with environmental variables

A significant positive correlation was found between absolute abundance of Vibrionaceae and water temperature in samples collected at LTER-C1 station (Spearman’s *ρ* = 0.62; *p* < 0.001). While we did not find any significant association between Vibrionaceae oligotypes and salinity or Chl *a*, a positive correlation emerged with POC concentration (Spearman’s *ρ* = 0.34; *p* < 0.05).

## Discussion

### Phylogenetic assignment and consensus-based approach

This study used the phylogenetic placement combined within a *consensus-based* approach to achieve a fine, species-level characterization of bacteria belonging to the Vibrionaceae family in different sites of the Northern Adriatic Sea.

Although we acknowledge that the term “species” is often inappropriate in the context of prokaryotes, particularly when referring to uncultured organisms, the use of the term “species” for bacterial groups of clinical relevance (including Vibrionaceae) is common practice (Oliver et al. 2012). Furthermore, in this work, we refer to the “species” level to highlight the potential ecological role or pathogenicity of the detected oligotypes.

Our analysis contributed to the identification of the components of bacterial blooms, and to detect potential human and animal pathogens.

Given the importance of the Vibrionaceae family in terms of human health and ecosystem functioning, a better understanding of its dynamics is fundamental. This is even more important in coastal areas, typically characterized by the presence of urbanization, aquaculture, industries, touristic pressure, sewage- and wastewater-related pollution.

Our workflow included the use of (*i*) control sequences, (*ii*) different reference databases, and (*iii*) the comparison between the phylogenetic placement and de novo oligotype phylogenies. This pipeline increased the reliability and the accuracy of the taxonomic assignment at the lower levels (i.e., “species”), when compared to the one obtained using only a reference database (e.g., SILVA) or using the phylogenetic placement alone. The phylogenetic placement algorithms require a reference tree, a corresponding reference multiple sequence alignment, and a collection of query sequences: the output is a set of assignments of the query sequences to branches of the tree (Matsen et al 2012). This tool has been recently used to finely characterize microbial communities in coastal marine (Wilson et al. 2021) and freshwater (Iniesto et al. 2021) environments. Moreover, it has been applied to profile the gut microbiota in toxic and non-toxic puffer fish species (Li et al. 2020) and to assign taxonomy to the white shrimp *Litopenaeus vannamei* gut oligotypes allowing to study the link between the presence and abundance of pathogenic opportunistic *Vibrio* species and atypical mass mortality (López-Cortés et al. 2020). Likewise, in our study, this tool allowed us to assign the vast majority of Vibrionaceae oligotypes at the species level (Fig. 2). The use of “long” and “short” control sequences previously obtained from the same sites permitted to estimate the effect of the length of the query sequences, a key factor in amplicon sequence data, and, at the same time, to help with their correct assignment.

The workflow we used included a step in which the taxonomy assigned by the phylogenetic placement was compared with the one obtained through different reference databases at the genus level (i.e., the lowest level reached by most amplicon sequence data), and some discrepancies emerged (Table S3). Disagreements in taxonomic assignments have been mostly attributed to differences in database size and release version, structure, annotation approach (Balvočiūtė and Huson 2017; Edgar 2018) (e.g., SILVA uses a phylogeny-based combination of automated and manual curation, RDP uses a Naïve Bayesian Classifier, BLAST uses local alignments), and updates in bacterial systematics, which affect also the Vibrionaceae family (Ast et al. 2009; Labella et al. 2017). In these cases, we tested and intersected the different approaches to achieve a *consensus-based* assignment. The removal of ambiguous oligotypes resulted in the removal of ~ 20% of the oligotypes in the final dataset. This step was crucial for reaching the lower taxonomic levels in the most accurate way; even if in our dataset, the ambiguous oligotypes represented a small portion in terms of relative abundance (~ 3%), in other datasets, the proportion could be higher and therefore we recommend including this comparison and selection in the analysis workflow.

Interestingly, the taxonomic assignment disagreement mostly affected only few species of Vibrionaceae: *Enterovibrio pacificus*, *Salinivibrio proteolyticus*, and *Paraphotobacterium marinum* (Table S4). Further investigations, considering broader datasets and/or mock communities, should be performed to test whether these taxa are consistently more prone than others to disagreements, for instance due to the lack of reference sequences, and/or incorrect, old, or spurious nomenclature (as suggested by Park and Won 2018). We could exclude that this was due to an under-representation of these taxa in the reference databases, as all were present except for *P. marinum* in GTDB ssu_r86.1_20180911 (https://osf.io/25djp/wiki/home/) and RDP (Cole et al. 2007).

Our workflow was designed to extract maximum information from datasets generated by 16S rRNA gene amplicon sequencing, the gold standard in the study of prokaryotic environmental communities (Yarza et al. 2014; Goodwin et al. 2017). However, additional markers can be used to increase the taxonomic resolution of targeted taxonomic groups. In Vibrionaceae, heat shock protein 60 (hsp60; Jesser and Noble 2018) has been successfully used and, in combination with the 16S rRNA gene, has provided useful information about the potential emergence of pathogens.

### Vibrionaceae communities in North Adriatic coastal sites

At the sampling sites investigated in the present study, Vibrionaceae family members generally represented less than 1% of the total microbial community (Figs. 3 and 4). It is commonly recognized that this family is generally low abundant in seawater microbial communities (Takemura et al. 2014; Fuhrman et al. 2015). However, despite their low relative abundance, members of this family have repeatedly demonstrated to be leading actors in organic matter dynamics, especially in marginal seas and in freshwater-influenced areas (Takemura et al. 2014; Vezzulli et al. 2016; Zhang et al. 2018). Phenotypic characterizations of culturable *Vibrio* representatives indicate that most members of this genus can degrade and utilize over 40 organic compounds (Farmer et al. 2005), including sugars (mono-, di-, and polysaccharides), alkanes, and lipids (Johnson 2013; Zhang et al. 2018).

**Fig. 6:**
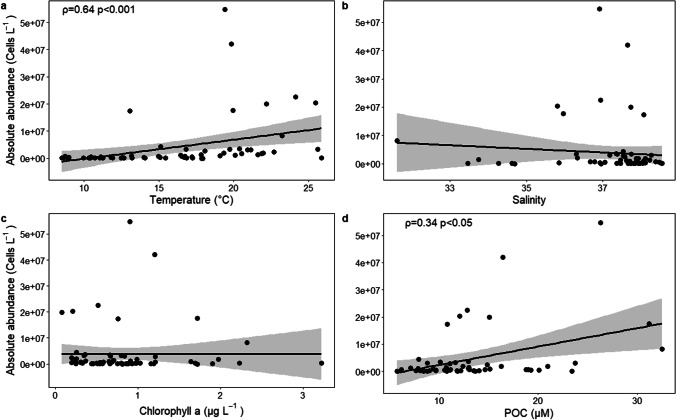
Scatterplots of the correlation (Spearman) between Vibrionaceae absolute abundance and (**a**) temperature, (**b**) salinity, (**c**) chlorophyll *a*, and (**d**) particulate organic carbon (POC) concentration. Spearman’s *ρ* and *p* values are shown only for significant correlations

Due to this capability of exploiting a wide array of substrates, Vibrionaceae representatives are able to form transient blooms in response to favorable environmental conditions, like micronutrients (e.g., following Saharan dust deposition events, Westrich et al. 2016) or organic matter pulses (e.g., phytoplankton or jellyfish blooms, Miller et al. 2005; Gilbert et al. 2012; Tinta et al., 2020). In our time-series, Vibrionaceae blooms were observed between June and October in both surface and bottom samples at the C1 station (Fig. 3) as already detected in coastal areas, including the northern Adriatic Sea (Gilbert et al. 2012; Tinta et al. 2015). Vibrionaceae diversity and abundance in water are known to be influenced by different biotic and abiotic factors (Kingsley 2014; Palit and Nair 2013). We found a significant positive correlation between Vibrionaceae abundance and temperature (Fig. 6), in line with growing observations that temperature is among the main drivers of Vibrionaceae dynamics in marine environments (Baker-Austin et al. 2018; Zhang et al. 2018; Wong et al. 2019).

While salinity is often regarded as an important factor in explaining the abundance patterns of Vibrionaceae family members (see Zhang et al. 2018 for a review), we did not identify a significant relationship with salinity in our dataset. A likely explanation for this lack of relationship would rely on the narrow salinity gradient at our sampling station (31.6–38.6 and 36.7–38.5 in surface and bottom samples, respectively). In fact, studies reporting salinity as an important factor in explaining Vibrionaceae dynamics are often conducted across transitional environments or in estuarine habitats, encompassing a much larger variability range (e.g., 0–27, Takemura et al. 2014).

Besides temperature, POC concentration represented a significant positive driver of Vibrionaceae abundance in samples in the C1 station. Members of this family are often found to be tightly associated with a variety of particulate substrates, from plastic fragments to phytoplankton cells (Hsieh et al. 2007; Kesy et al. 2021; Sampaio et al. 2022). Experimental and genomic investigations of their metabolic capabilities have highlighted the presence of an advanced exoenzymatic machinery for polysaccharide degradation (Zhang et al. 2018). Therefore, given the significative presence of carbohydrates in organic particles (Kharbush et al. 2020), it is likely that they represent an important nutritional source for members of Vibrionaceae.

Time-series analyses (also in the Gulf of Trieste; Celussi and Cataletto 2007; Tinta et al. 2015) showed that species of this family can occasionally bloom, even becoming the prevalent members of the bacterial community for a short period of time or several weeks, before declining (Vergin et al. 2013; Fuhrman et al. 2015).

At the sampled marine sites, Vibrionaceae blooms were mainly formed by two species, identified by the phylogenetic placement as *V. chagasii* and *V. owensii* (the latter found only in C1 samples, Fig. 3 and 4), in agreement with previous observations that identified few Vibrionaceae oligotypes “blooming” in response to changes in environmental conditions (Gilbert et al. 2012).

*V. chagasii* is found to be ubiquitous in aquatic environments (Thomson et al. 2004) as well as among the components of molluscan microbiomes (Romalde et al. 2014). Although its pathogenicity has not been clarified yet, several studies point out its capability to infect marine invertebrates (Teng et al. 2012; Liang et al. 2019) and fishes (Fabbro et al. 2012). The ecological role of this bacterium in the pelagic environment is still poorly understood, but further information is expected to be acquired due to the recent sequencing of its full genome (Sanches-Fernandes et al. 2021). *V. owensii* (synonym *V.* *communis*) belongs to the Harveyi clade (Cano-Gomez et al. 2011). It represents one of the four species of the group (together with *V. harveyi*, *V. campbellii*, and *V. rotiferianus*) acknowledged as pathogens in farmed fish, shellfish, and crustaceans (Goulden et al. 2012; Liu et al. 2018; Pretto 2020), as well as a putative coral pathogen (Amin et al. 2016).

In our dataset, among pathogenic species (that, apart from *V. owensii*, were present in very low abundances, Fig. 3), we detected *V. anguillarum*, known to cause vibriosis in fish and shellfish and frequently retrieved in marine and estuarine environments (Kim et al. 2012; Ma et al. 2017; Gao et al. 2018) and *Vibrio fluvialis*, commonly found in coastal environments and considered an emerging human foodborne pathogen (Ramamurthy et al. 2014), in all sites. Other identified species were *Photobacterium damselae*, a free-living animal pathogen that can be found in coastal environments (Novianty and Budiarti 2014; Terceti et al. 2018), and *Vibrio tapetis*, causing brown ring disease in various bivalve species and other vibriosis in fishes (Gomez-Gil et al. 2014). *V. parahaemolyticus*, identified in the northern Adriatic coastal waters with biochemical and molecular-based methodologies (Fabbro et al. 2010) if present, was probably in abundances too low to be detected with amplicon sequencing.

Together with the high biodiversity of the coastal zone of the Gulf of Trieste (Nasi et al. 2017), we speculate that the presence of animal pathogens could also be due to the abundant mussel farms in the area (Franzo et al. 2014), which could represent a potential reservoir and source of molluscs-associated Vibrionaceae.

### Depuration plants’ Vibrionaceae

Our investigation of the Vibrionaceae family revealed a wide taxonomical and phylogenetic diversity in the three marine sampling sites, in contrast with low numbers of oligotypes retrieved in the treated wastewater samples (Fig. 5). In the same way, the Vibrionaceae relative abundances in the treated sewage were much lower than in seawater samples, especially in the case of San Giorgio DP. Although the treated sewage from the two DP shared a few Vibrionaceae species (*Vibrio chagasii*, *V. orientalis*, *Photobacterium swingsii*), they differed in terms of community structure (Fig. S1). Notably, only a few species retrieved in DP samples were also found at sea, in the proximity of the respective pipeline outfalls. This, together with the low number of retrieved reads, suggests that DP may represent (if any) only a minor source of Vibrionaceae for the surrounding marine coastal environment. The two DP investigated in this study provided for final disinfection prior to unloading the treated sewage in the outlet pipeline. Similarly, in a recent investigation in the central Adriatic Sea, we found that DP contributed only partially to the pool of Vibrionaceae retrieved at sea (next to their outfalls) independently on the presence/absence of processes aimed at decreasing the microbiological load, such as disinfection or activated sludge treatments (Fonti et al. 2021).

Lignano Sabbiadoro DP showed the presence, in very low abundance, of *Vibrio cholerae* and of *V. navarrensis*, which we never detected in seawater. *Vibrio cholerae*, besides being autochthonous in aquatic environment, finds favorable survival and growth conditions in oligohaline, organic matter-rich, neutral to alkaline sewage (Takemura et al. 2014; Baron et al. 2017). Among the over 200 O serogroups of this species, only two strains (O1 and O139) are linked to severe disease and cholera (Momba and Azab El-Liethy 2017). Even if our analysis could not lead to the identification of the serogroup, we can exclude the presence of the cholera disease-associated strains, as the vast majority of the forms of *V. cholerae* detected in the environment are harmless estuarine bacteria (Rivera et al. 2001). *Vibrio navarrensis* has been found to be associated with low salinity condition in Thyrrenian coastal environment (Matteucci et al. 2015). Firstly isolated in sewage (Urdaci et al. 1991), and later also in human clinical specimens (Gladney et al. 2014; Schwartz et al. 2017), it is considered a rare human pathogen, characterized by distinctive molecular makers and virulence-associated genes (Lee and Raghunath 2018).

Interestingly, the most abundant species found in treated wastewater samples (*V. chagasii*, *P. swingsii*, *V. anguillarum*) are of marine origins, being carried into the plants by seawater intrusion (N. De Bortoli, pers. comm.).

Taken together, our results highlight that, differently from other potential pathogens (Fonti et al. 2021), members of the Vibrionaceae family retrieved at sea are unlikely to be related to wastewater inputs. Therefore, we suggest to carefully consider the inclusion of the genus *Vibrio* in the “blacklist” of potentially pathogenic genera in surveys of microbiological pollution in coastal waters (e.g., Paliaga et al. 2017; Fonti et al. 2021; Orel et al. 2022), also in light of the fact that the distribution of pathogenic traits within a single species (e.g., *V. parahaemolyticus*, *V. vulnificus*) carried by specific genes (e.g., *tdh*, *trh*, *vcgC*) is highly variable (Fabbro et al. 2010; Nigro et al. 2022).

## Conclusions

Our workflow proved to be useful for a fine-level biodiversity analysis of specific target organisms in environmental studies based on amplicon sequencing. This approach represents a further step toward building a shared, updated, and curated phylogeny of the Vibrionaceae family that could be applied to new and old data for a continuous improvement of molecular tools (Kublanovskaya et al. 2019; Roush et al. 2021). It allows the capitalization of data generated at LTER sites or within genomic observatories that use 16S rRNA to study the prokaryotic communities. The construction of smaller, *ad hoc* reference databases has been proved useful in enhancing the 16S rRNA gene amplicon sequencing taxonomic resolution, reducing the massive size of the search space, and lowering competition among similar DNA sequences (Ritari et al. 2015).

In the near future, the third generation of sequencing platforms such as Pacific Biosciences (Rhoads and Au 2015) and Oxford Nanopore Technologies (Jain et al. 2018) that allow for longer (up to thousands kb) read length will become more and more common. Even if their error rate is still high (Fu et al. 2019), their contribution will be useful in the perspective of constructing consensus sequences covering the full length of barcode regions, combining short and long reads.

Even if our study was focused on seawater, our approach is also applicable to other target matrices such as sediment and marine metazoan microbiomes (e.g., fish gut), where Vibrionaceae play a significant role.

Access to reliable and fine-level taxonomic data is essential not only for the assessment of the diversity and the composition of the microbial communities, but also for ecological research. The possibility to link specific taxonomic groups with biological, chemical, and physical parameters is fundamental to understand the behavior and the response of microbial assemblages to environmental changes.

## Supplementary Information

Below is the link to the electronic supplementary material.

Supplementary file1 (DOCX 712 KB)

## Data Availability

The sequences generated for this study can be found in the Sequence Reads Archive (SRA) at NCBI under the accession numbers PRJNA767222, PRJNA818117, PRJNA818144, PRJNA818839, and PRJNA818858.
