# Supplementary material for: Improving environmental monitoring of Vibrionaceae in coastal ecosystems through 16S rRNA gene amplicon sequencing
Source: Environ Sci Pollut Res Int. 2022 Sep 2;29(44):67466–82. doi: 10.1007/s11356-022-22752-z (PMC9492620; doi:10.1007/s11356-022-22752-z)
Supplement: Supplementary file 1 — Supplementary file1 (DOCX 712 KB) [file 11356_2022_22752_MOESM1_ESM.docx]

**Supplementary information**

**Improving environmental monitoring of Vibrionaceae in coastal ecosystem through amplicon sequencing**

Elisa Banchi*, Vicenzo Manna, Viviana Fonti, Cinzia Fabbro, Mauro Celussi*

National Institute of Oceanography and Applied Geophysics - OGS, Via A. Piccard, 54, 34151 Trieste, Italy

***Correspondence:**

email: ebanchi@ogs.it; tel: +39 0402140721; fax: +39 0402249770

email: mcelussi@ogs.it; tel: +39 0402140732; fax: +39 0402249770


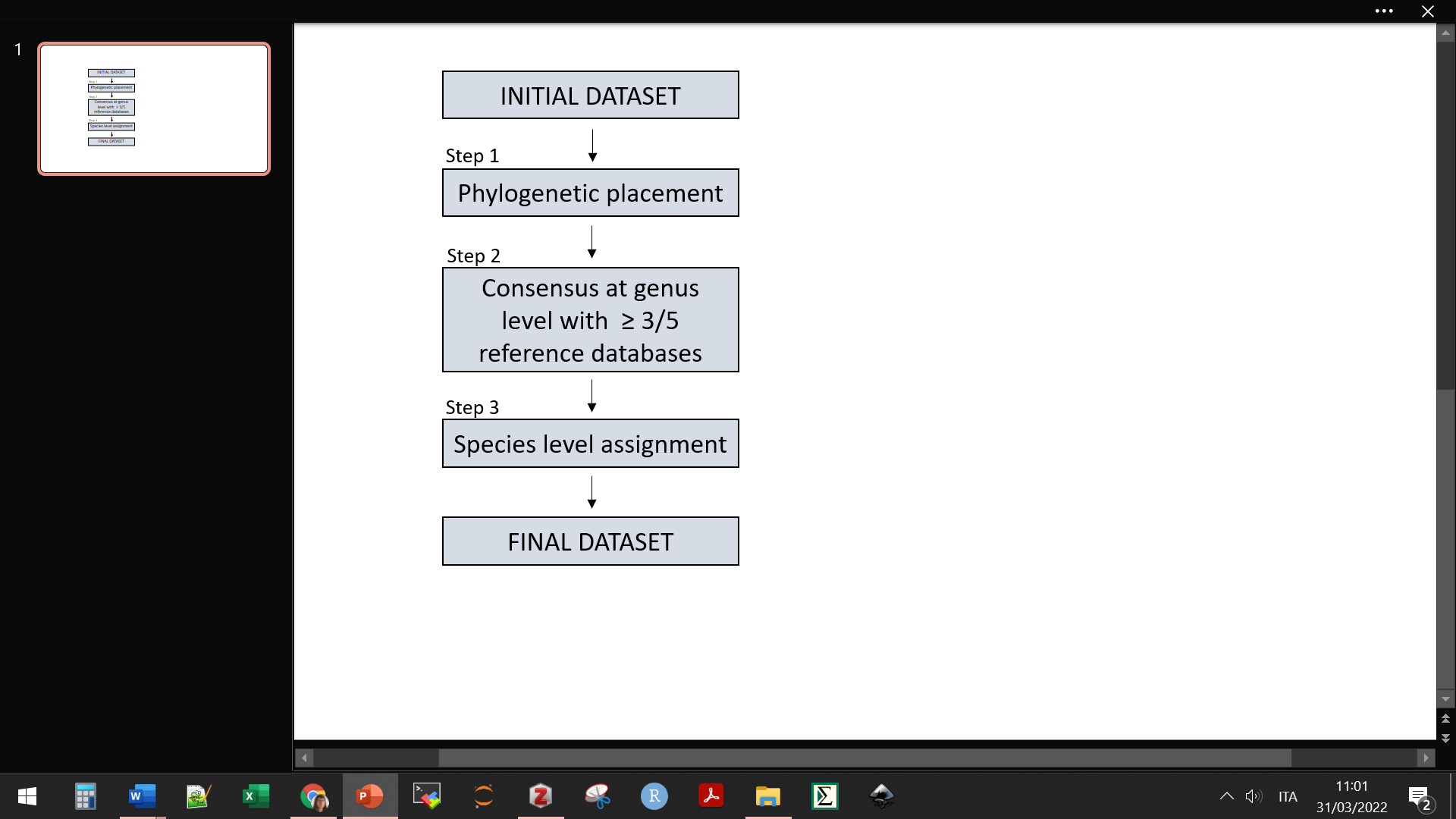


**Fig. S1** *Consensus-based* workflow used for the species-level assignment of the Vibrionaceae oligotypes.

**
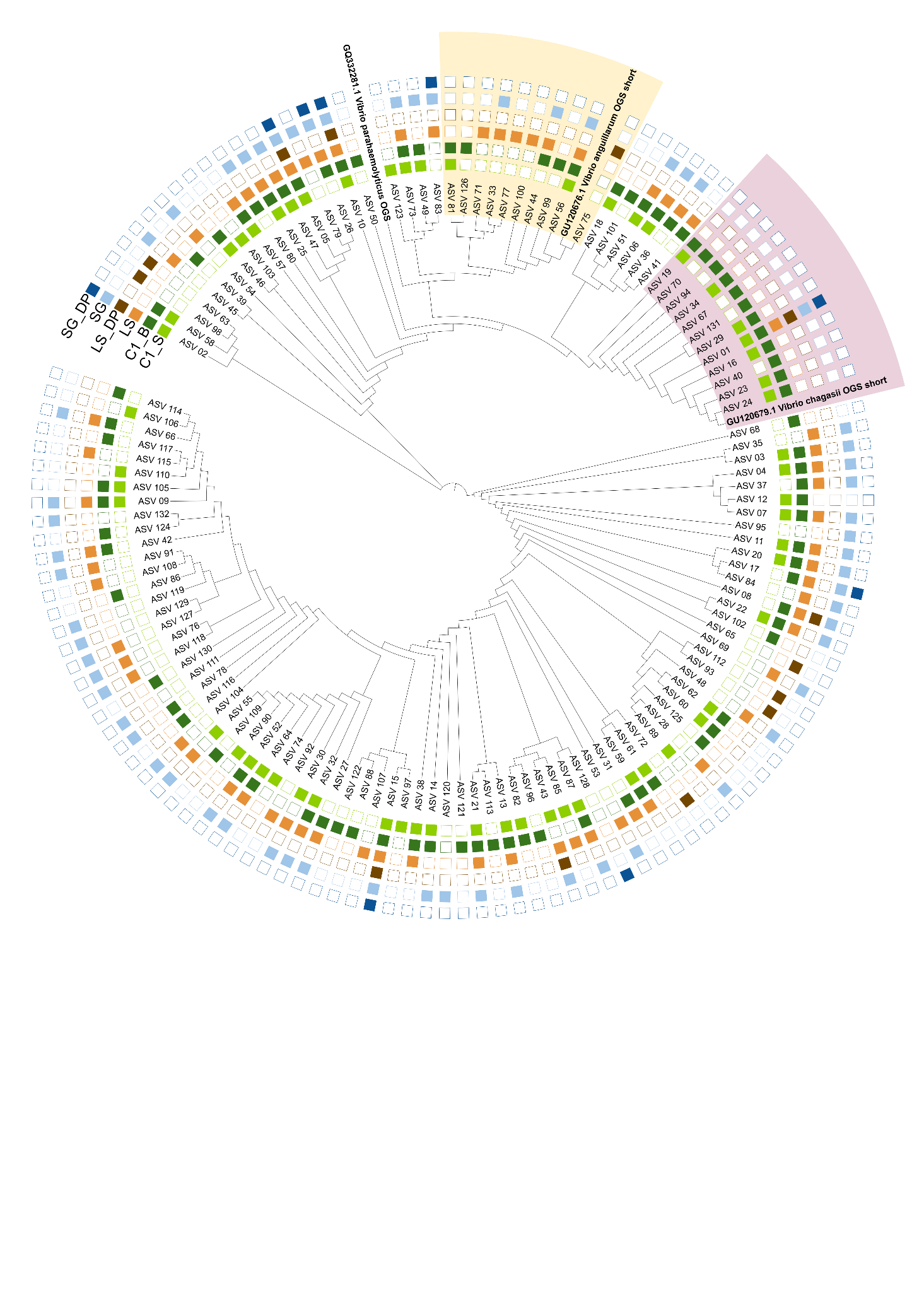
**

**Fig. S2** RAxML Maximum likelihood phylogeny of the Vibrionaceae oligotypes including the control sequencing (in bold). The presence/absence of each oligotype in the datasets are shown in the outer circles. DP: depurator

**Table S1.** List of the Vibrionaceae NCBI accession numbers used to build the reference tree

| AB000278.1 Photobacterium iliopiscarium | AM279734.1 Salinivibrio sharmensis | GU018180.2 VIbrio owensii | X74710.1 Vibrio mediterranei |
| --- | --- | --- | --- |
| AB000390.2 Vibrio halioticoli | AM942722.1 Enterovibrio nigricans | GU065210.1 Photobacterium jeanii | X74711.1 Vibrio metschnikovii |
| AB013297.1 Vibrio rumoiensis | AY155585.1 Vibrio supersteus | GU078672.1 Vibrio owensii | X74713.1 Vibrio mimicus |
| AB016982.1 Photobacterium indicum | AY254040.1 Vibrio hispanicus | GU223601.1 Vibrio caribbeanicus | X74714.1 Vibrio natriegens |
| AB032015.1 Photobacterium damselae | AY257972.1 Vibrio gallicus | GU929924.1 Vibrio variabilis | X74715.1 Vibrio navarrensis |
| AB159513.1 Photobacterium indicum | AY341439.1 Photobacterium kishitanii | GU929925.1 Vibrio maritimus | X74716.1 Vibrio nereis |
| AB285018.1 Salinivibrio siamensis | AY426979.2 Vibrio neonatus | HE573748.1 Grimontia indica | X74717.1 Vibrio nigripulchritudo |
| AB428873.1 Photobacterium aquimaris | AY426980.1 Vibrio ezurae | HM452945.2 Photobacterium atrarenae | X74718.1 Vibrio ordalii |
| AB428897.1 Vibrio azureus | AY538749.1 Photobacterium frigidiphilum | JF316656.1 Vibrio alfacsensis | X74719.1 Vibrio orientalis |
| AB428909.1 Vibrio sagamiensis | AY551089.1 Photobacterium halotolerans | JF931139.1 Vibrio zhanjiangensis | X74722.1 Vibrio pelagius |
| AB464964.1 Aliivibrio sifiae | AY554009.1 Photobacterium lipolyticum | JQ993843.1 Candidatus Photodesmus blepharus | X74723.1 Vibrio proteolyticus |
| AB562589.1 Vibrio jasicida | AY643710.1 Photobacterium iliopiscarium | KF360256.1 Candidatus Photodesmus katoptron | X74724.1 Vibrio splendidus |
| AF057016.1 Salinivibrio vallismortis | AY750575.1 Vibrio harveyi | KU315478.1 Photobacterium alginatilyticum | X74725.1 Vibrio tubiashi |
| AF118021.1 Enterovibrio calviensis | AY781193.1 Photobacterium aplysiae | LT821387.1 Salinivibrio socompensis | X76333.1 Vibrio vulnificus |
| AF124055.3 Vibrio aerogenes | AY960847.2 Photobacterium ganghwense | MW404042.1 Grimontia sedimenti | X76335.1 Vibrio fluvialis |
| AF388386.1 Vibrio parahaemolyticus | D21226.1 Photobacterium profundum | NR 104870.1 Catenococcus thiocycli | X76336.1 Vibrio furnissii |
| AF462458.1 Vibrio ruber | D25307.1 Photobacterium angustum | NR 116862.1 Grimontia marina | X76337.1 Vibrio cholerae |
| AJ132227.1 Aliivibrio wodanis | D25310.1 Photobacterium phosphoreum | NR 125679.1 Photobacterium piscicola | X78105.1 Photobacterium damselae |
| AJ278881.1 Vibrio lentus | DQ092443.1 Salinivibrio proteolyticus | NR 126301.1 Photobacterium sanctipauli | X95527.1 Salinivibrio costicola |
| AJ293802.1 Vibrio pelagius | DQ097523.1 Vibrio litoralis | NR 132335.1 Echinimonas agarilytica | X99761.1 Vibrio mytili |
| AJ310647.1 Vibrio agarivorans | DQ451211.1 Vibrio sinaloensis | NR 133050.1 Photobacterium marinum | X99762.2 Vibrio diabolicus |
| AJ316171.1 Vibrio neptunius | DQ481610.1 Vibrio cyclitrophicus | NR 133814.1 Photobacterium aestuarii | Y08430.1 Vibrio tapetis |
| AJ316172.1 Vibrio brasiliensis | DQ534014.1 Photobacterium lutimaris | NR 133815.1 Photobacterium aquae | Y13830.1 Vibrio pectenicida |
| AJ316178.1 Vibrio hispanicus | DQ847123.1 Vibrio rhizosphaerae | NR 136855.1 Photobacterium panuliri |  |
| AJ316181.1 Vibrio xuii | DQ914239.1 Vibrio rarus | NR 145878.1 Photobacterium galatheae |  |
| AJ316187.1 Vibrio rotiferatus | DQ922915.1 Vibrio comitans | NR 146675.1 Photobacterium sanguinicancri |  |
| AJ316192.1 Vibrio tasmaniensis | DQ922920.1 Vibrio inusitatus | NR 147757.1 Enterovibrio pacificus |  |
| AJ316193.1 Vibrio kanaloae | EF094887.1 Vibrio crassostreae | NR 151922.1 Grimontia celer |  |
| AJ316194.1 Vibrio pacinii | EF094888.1 Vibrio gigantis | NR 151928.1 Paraphotobacterium marinum |  |
| AJ316199.1 Vibrio chagasii | EF488079.1 Vibrio porteresiae | NR 156814.1 Photobacterium carnosum |  |
| AJ316208.1 Enterovibrio norvegicus | EF583688.1 Salinivibrio budaii | NR 157004.1 Photobacterium proteolyticum |  |
| AJ345063.1 Vibrio probioticus | EF599161.2 Vibrio breoganii | NR 157685.1 Salinivibrio kushneri |  |
| AJ421445.1 Vibrio ichthyoenteri | EF599162.1 Vibrio celticus | NR 163605.1 Thaumasiovibrio subtropicus |  |
| AJ437191.1 Vibrio penaeicida | EF599163.1 Vibrio atlanticus | NR 171499.1 Photobacterium chitinilyticum |  |
| AJ437192.1 Vibrio ichthyoenteri | EU082035.1 Vibrio hangzhouensis | U46579.1 Vibrio scophthalmi |  |
| AJ437616.1 Aliivibrio logei | EU143360.1 Vibrio areninigrae | X16895.1 Vibrio anguillarum |  |
| AJ440005.1 Vibrio corallilyticus | EU144014.1 Vibrio mangrovi | X56575.1 Vibrio campbellii |  |
| AJ491290.1 Vibrio pomeroyi | EU185839.1 Aliivibrio thorii | X56576.1 Vibrio alginolyticus |  |
| AJ514909.1 Grimontia hollisae | EU541604.1 Aliivibrio finisterrensis | X56580.1 Vibrio parahaemolyticus |  |
| AJ514916.1 Vibrio fortis | EU541605.1 Vibrio gallaecicus | X70643.1 Aliivibrio salmonicida |  |
| AJ515230.1 Vibrio splendidus | FJ009624.1 Vibrio atypicus | X74686.1 Photobacterium leiognathi |  |
| AJ582810.1 Vibrio gigantis | FJ968722.1 Vibrio casei | X74689.1 Vibrio aestuarianus |  |
| AJ630103.1 Vibrio ponticus | FN421434.1 Vibrio hippocampi | X74698.1 Vibrio cincinnatiensis |  |
| AJ640132.1 Salinivibrio costicola | FN687911.1 Vibrio jasicida | X74699.1 Salinivibrio costicola |  |
| AJ842343.1 Enterovibrio coralii | FN796493.1 Photobacterium aphoticum | X74701.1 Vibrio diazotrophicus |  |
| AJ842344.1 Photobacterium rosenbergii | GQ260188.1 Photobacterium gaetbulicola | X74702.1 Aliivibrio fischeri |  |
| AJ845017.1 Vibrio aestuarianus | GQ281380.1 Vibrio stylophorae | X74703.1 Vibrio fluvialis |  |
| AM162656.1 Vibrio cyclitrophicus | GQ352641.1 Vibrio plantisponsor | X74705.1 Vibrio gazogenes |  |
| AM162657.1 Vibrio kanaloae | GQ386822.1 Photobacterium swingsii | X74706.1 Vibrio harveyi |  |
| AM235737.1 Vibrio anguillarum | GQ397859.1 Vibrio xiamenensis | X74708.1 Aliivibrio logei |  |

**Table S2.** Phylogenetic placement and respective numbers of Vibrionaceae oligotypes.

|  | n. |  | n. |
| --- | --- | --- | --- |
| *Aliivibrio fischeri* | 2 | *Thaumasiovibrio subtropicus* | 3 |
| *Aliivibrio sp.* | 1 | *Vibrio anguillarum* | 11 |
| *Candidatus Photodesmus* | 6 | *Vibrio anguillarum/ordalii* | 1 |
| *Enterovibrio calviensis* | 1 | *Vibrio atypicus* | 1 |
| *Enterovibrio coralii* | 4 | *Vibrio caribbeanicus* | 6 |
| *Enterovibrio norvegicus* | 1 | *Vibrio chagasii* | 8 |
| *Enterovibrio pacificus* | 6 | *Vibrio cholerae* | 1 |
| *Grimontia marina* | 3 | *Vibrio cholerae/mimicus* | 1 |
| *Grimontia sp.* | 2 | *Vibrio diazotrophicus* | 1 |
| *Paraphobacterium marinum* | 6 | *Vibrio estuarianus* | 1 |
| *Photobacterium aestuarii* | 1 | *Vibrio fluvialis* | 2 |
| *Photobacterium angustum* | 2 | *Vibrio hispanicus/areninigrae* | 2 |
| *Photobacterium aphoticum* | 5 | *Vibrio inusitatus* | 1 |
| *Photobacterium atrarenae* | 1 | *Vibrio ippocampi* | 4 |
| *Photobacterium damselae* | 3 | *Vibrio kanaloae/splendidus/lentus* | 1 |
| *Photobacterium gaetbulicola* | 1 | *Vibrio navarrensis* | 2 |
| *Photobacterium lutimaris* | 1 | *Vibrio orientalis* | 1 |
| *Photobacterium marinum* | 1 | *Vibrio owensii* | 1 |
| *Photobacterium panuliri* | 1 | *Vibrio pectenicida/tapetis* | 4 |
| *Photobacterium rosenbergii* | 2 | *Vibrio ponticus* | 1 |
| *Photobacterium sanctipauli* | 1 | *Vibrio sp.* | 11 |
| *Photobacterium sanguinicancri* | 2 | *Vibrio sp. (cholerae clade)* | 1 |
| *Photobacterium sp.* | 3 | *Vibrio sp. (diazotrophicus clade)* | 1 |
| *Photobacterium swingsii* | 1 | *Vibrio splendidus* | 2 |
| *Photobacterium swingsii/sanguinicancri* | 1 | *Vibrio xiamenensis* | 2 |
| *Salinivibrio proteolyticus* | 4 |  |  |

**Table S3.** Taxonomic assignment of each oligotypes and selection for the final dataset. PP: phylogenetic placement

| **Oligotype** | **PP Species** | **PP Genus** | **SILVA** | **SILVA LCA** | **RDP** | **BLAST** | **GTDB** | **Final Dataset** |
| --- | --- | --- | --- | --- | --- | --- | --- | --- |
| ASV_01 | *Vibrio chagasii* | Vibrio | Vibrio | Vibrio | Vibrio | Vibrio | Vibrio | Yes |
| ASV_02 | *Vibrio sp.* | Vibrio | Vibrionaceae | Vibrio | Unclassified | Vibrio | Vibrio | No |
| ASV_03 | *Vibrio sp.* | Vibrio | Vibrio | Vibrio | Vibrio | Vibrio | Vibrio | No |
| ASV_04 | *Vibrio owensii* | Vibrio | Vibrio | Vibrio | Vibrionaceae | Vibrio | Vibrio | Yes |
| ASV_05 | *Vibrio pectenicida/tapetis* | Vibrio | Vibrio | Vibrio | Vibrio | Vibrio | Vibrio | Yes |
| ASV_06 | *Vibrio kanaloae/splendidus/lentus* | Vibrio | Aliivibrio | Vibrio | Vibrio | Vibrio | Vibrionaceae | Yes |
| ASV_07 | *Photobacterium angustum* | Photobacterium | Catenococcus | Photobacterium | Vibrionaceae | Photobacterium | Photobacterium | Yes |
| ASV_08 | *Vibrio caribbeanicus* | Vibrio | Vibrio | Vibrio | Vibrio | Vibrio | Vibrio | Yes |
| ASV_09 | *Enterovibrio norvegicus* | Enterovibrio | Enterovibrio | Enterovibrio | Enterovibrio | Enterovibrio | Enterovibrio | Yes |
| ASV_10 | *Vibrio orientalis* | Vibrio | Vibrio | Vibrio | Vibrio | Vibrio | Vibrio | Yes |
| ASV_11 | *Vibrio caribbeanicus* | Vibrio | Vibrio | Vibrio | Vibrio | Vibrio | Vibrio | Yes |
| ASV_12 | *Photobacterium angustum* | Photobacterium | Vibrionaceae | Photobacterium | Vibrionaceae | Photobacterium | Photobacterium | Yes |
| ASV_13 | *Photobacterium damselae* | Photobacterium | Photobacterium | Photobacterium | Photobacterium | Photobacterium | Photobacterium | Yes |
| ASV_14 | *Photobacterium sp.* | Photobacterium | Photobacterium | Photobacterium | Photobacterium | Photobacterium | Photobacterium | No |
| ASV_15 | *Photobacterium swingsii* | Photobacterium | Photobacterium | Photobacterium | Photobacterium | Photobacterium | Photobacterium | Yes |
| ASV_16 | *Candidatus Photodesmus sp.* | Candidatus Photodesmus | Vibrio | Vibrio | Vibrio | Vibrio | Vibrio | No |
| ASV_17 | *Vibrio caribbeanicus* | Vibrio | Vibrio | Vibrio | Vibrio | Vibrio | Vibrio | Yes |
| ASV_18 | *Vibrio splendidus* | Vibrio | Vibrio | Vibrio | Vibrio | Vibrio | Vibrio | Yes |
| ASV_19 | *Candidatus Photodesmus sp.* | Candidatus Photodesmus | Vibrio | Vibrio | Vibrio | Vibrio | Vibrio | No |
| ASV_20 | *Vibrio caribbeanicus* | Vibrio | Vibrio | Vibrio | Vibrio | Vibrio | Vibrio | Yes |
| ASV_21 | *Photobacterium damselae* | Photobacterium | Vibrionaceae | Vibrio | Vibrionaceae | Vibrio | Photobacterium | No |
| ASV_22 | *Photobacterium aphoticum* | Photobacterium | Vibrio | Vibrio | Vibrio | Vibrio | Vibrio | No |
| ASV_23 | *Vibrio chagasii* | Vibrio | Vibrio | Vibrio | Vibrio | Vibrio | Vibrio | Yes |
| ASV_24 | *Candidatus Photodesmus sp.* | Candidatus Photodesmus | Vibrio | Vibrio | Vibrio | Vibrio | Vibrio | No |
| ASV_25 | *Vibrio estuarianus* | Vibrio | Vibrio | Vibrio | Vibrio | Vibrio | Vibrio | Yes |
| ASV_26 | *Candidatus Photodesmus sp.* | Candidatus Photodesmus | Vibrio | Vibrio | Vibrio | Vibrio | Vibrio | No |
| ASV_27 | *Photobacterium sanguinicancri* | Photobacterium | Photobacterium | Photobacterium | Photobacterium | Photobacterium | Photobacterium | Yes |
| ASV_28 | *Vibrio ippocampi* | Vibrio | Vibrio | Vibrio | Vibrionaceae | Vibrio | Vibrio | Yes |
| ASV_29 | *Vibrio chagasii* | Vibrio | Vibrio | Vibrio | Vibrio | Vibrio | Vibrio | Yes |
| ASV_30 | *Photobacterium_atrarenae* | Photobacterium | Photobacterium | Photobacterium | Photobacterium | Photobacterium | Photobacterium | Yes |
| ASV_31 | *Vibrio fluvialis* | Vibrio | Vibrio | Vibrio | Vibrio | Vibrio | Vibrio | Yes |
| ASV_32 | *Photobacterium sanctipauli* | Photobacterium | Photobacterium | Photobacterium | Photobacterium | Photobacterium | Photobacterium | Yes |
| ASV_33 | *Vibrio anguillarum* | Vibrio | Vibrio | Vibrio | Vibrio | Vibrio | Vibrio | Yes |
| ASV_34 | *Candidatus Photodesmus sp.* | Candidatus Photodesmus | Vibrio | Vibrio | Vibrio | Vibrio | Vibrio | No |
| ASV_35 | *Vibrio sp.* | Vibrio | Vibrio | Vibrio | Unclassified | Vibrio | Vibrio | No |
| ASV_36 | *Vibrio splendidus* | Vibrio | Aliivibrio | Vibrio | Vibrio | Vibrio | Vibrionaceae | Yes |
| ASV_37 | *Photobacterium aphoticum* | Photobacterium | Photobacterium | Vibrio | Photobacterium | Vibrio | Vibrio | No |
| ASV_38 | *Photobacterium sp.* | Photobacterium | Photobacterium | Photobacterium | Photobacterium | Photobacterium | Photobacterium | No |
| ASV_39 | *Vibrio sp.* | Vibrio | Vibrio | Vibrio | Vibrio | Vibrio | Vibrio | No |
| ASV_40 | *Vibrio chagasii* | Vibrio | Vibrio | Vibrio | Vibrio | Vibrio | Vibrio | Yes |
| ASV_41 | *Aliivibrio sp.* | Aliivibrio | Aliivibrio | Aliivibrio | Aliivibrio | Aliivibrio | Aliivibrio | No |
| ASV_42 | *Enterovibrio calviensis* | Enterovibrio | Enterovibrio | Enterovibrio | Enterovibrio | Enterovibrio | Enterovibrio | Yes |
| ASV_43 | *Salinivibrio proteolyticus* | Salinivibrio | Vibrionaceae | Unclassified | Unclassified | Vibrio | Thaumasiovibrio | No |
| ASV_44 | *Vibrio anguillarum/ordalii* | Vibrio | Vibrio | Vibrio | Vibrio | Vibrio | Vibrio | Yes |
| ASV_45 | *Vibrio sp.* | Vibrio | Vibrio | Vibrio | Vibrio | Vibrio | Vibrio | No |
| ASV_46 | *Vibrio ponticus* | Vibrio | Vibrio | Vibrio | Vibrionaceae | Vibrio | Vibrio | Yes |
| ASV_47 | *Vibrio pectenicida/tapetis* | Vibrio | Vibrio | Vibrio | Vibrio | Vibrio | Vibrio | Yes |
| ASV_48 | *Vibrio ippocampi* | Vibrio | Vibrio | Vibrio | Vibrionaceae | Vibrio | Vibrio | Yes |
| ASV_49 | *Vibrio hispanicus* | Vibrio | Vibrio | Vibrio | Vibrio | Vibrio | Vibrio | Yes |
| ASV_50 | *Vibrio sp.* | Vibrio | Vibrio | Vibrio | Vibrio | Vibrio | Vibrio | No |
| ASV_51 | *Aliivibrio fischeri* | Aliivibrio | Aliivibrio | Aliivibrio | Aliivibrio | Aliivibrio | Aliivibrio | Yes |
| ASV_52 | *Photobacterium gaetbulicola* | Photobacterium | Photobacterium | Photobacterium | Photobacterium | Photobacterium | Photobacterium | Yes |
| ASV_53 | *Vibrio fluvialis* | Vibrio | Vibrio | Vibrio | Vibrio | Vibrio | Vibrio | Yes |
| ASV_54 | *Vibrio sp.* | Vibrio | Catenococcus | Vibrio | Vibrionaceae | Vibrio | Vibrio | No |
| ASV_55 | *Thaumasiovibrio subtropicus* | ThaumasioVibrio | ThaumasioVibrio | Vibrionaceae | Vibrionaceae | ThaumasioVibrio | Thaumasiovibrio | Yes |
| ASV_56 | *Vibrio anguillarum* | Vibrio | Vibrio | Vibrio | Vibrio | Vibrio | Vibrio | Yes |
| ASV_57 | *Vibrio atypicus* | Vibrio | Vibrio | Vibrio | Vibrio | Vibrionaceae | Vibrio | Yes |
| ASV_58 | *Vibrio sp.* | Vibrio | Vibrio | Vibrio | Vibrionaceae | Vibrio | Vibrio | No |
| ASV_59 | *Vibrio inusitatus* | Vibrio | Vibrio | Vibrio | Vibrio | Vibrio | Vibrio | Yes |
| ASV_60 | *Photobacterium aphoticum* | Photobacterium | Vibrionaceae | Vibrionaceae | Vibrionaceae | Photobacterium | Photobacterium | Yes |
| ASV_61 | *Vibrio sp.* | Vibrio | Vibrio | Vibrio | Vibrio | Vibrio | Vibrio | No |
| ASV_62 | *Photobacterium aphoticum* | Photobacterium | Photobacterium | Vibrionaceae | Vibrionaceae | Photobacterium | Photobacterium | Yes |
| ASV_63 | *Vibrio navarrensis* | Vibrio | Vibrio | Vibrio | Vibrio | Vibrio | Vibrio | Yes |
| ASV_64 | *Photobacterium rosenbergii* | Photobacterium | Photobacterium | Photobacterium | Photobacterium | Photobacterium | Photobacterium | Yes |
| ASV_65 | *Vibrio diazotrophicus* | Vibrio | Vibrio | Vibrio | Vibrio | Vibrio | Vibrio | Yes |
| ASV_66 | *Paraphotobacterium marinum* | Paraphotobacterium | Vibrionaceae | Vibrionaceae | Unclassified | Photobacterium | Vibrionaceae | No |
| ASV_67 | *Candidatus Photodesmus sp.* | Candidatus Photodesmus | Vibrio | Vibrio | Vibrio | Vibrio | Vibrio | No |
| ASV_68 | *Vibrio sp.* | Vibrio | Vibrio | Vibrio | Vibrio | Vibrio | Vibrio | No |
| ASV_69 | *Vibrio sp. (diazotrophicus clade)* | Vibrio | Vibrio | Vibrio | Vibrio | Vibrio | Vibrio | Yes |
| ASV_70 | *Vibrio chagasii* | Vibrio | Vibrio | Vibrio | Vibrio | Vibrio | Vibrio | Yes |
| ASV_71 | *Vibrio anguillarum* | Vibrio | Vibrio | Vibrio | Vibrio | Vibrio | Vibrio | Yes |
| ASV_72 | *Vibrio sp. (cholerae clade)* | Vibrio | Vibrio | Vibrio | Vibrio | Vibrio | Vibrio | Yes |
| ASV_73 | *Vibrio pectenicida* | Vibrio | Vibrio | Vibrio | Vibrio | Vibrio | Vibrio | Yes |
| ASV_74 | *Photobacterium rosenbergii* | Photobacterium | Photobacterium | Photobacterium | Photobacterium | Photobacterium | Photobacterium | Yes |
| ASV_75 | *Vibrio anguillarum* | Vibrio | Vibrio | Vibrio | Vibrio | Vibrio | Vibrio | Yes |
| ASV_76 | *Enterovibrio coralii* | Enterovibrio | Enterovibrio | Enterovibrio | Enterovibrio | Enterovibrio | Enterovibrio | Yes |
| ASV_77 | *Vibrio anguillarum* | Vibrio | Vibrio | Vibrio | Vibrio | Vibrio | Vibrio | Yes |
| ASV_78 | *Paraphotobacterium marinum* | Paraphotobacterium | Enterovibrio | Vibrionaceae | Vibrionaceae | Enterovibrio | Vibrionaceae | No |
| ASV_79 | *Vibrio sp.* | Vibrio | Vibrio | Vibrio | Vibrio | Vibrio | Vibrio | No |
| ASV_80 | *Vibrio sp.* | Vibrio | Vibrio | Vibrio | Vibrio | Vibrio | Vibrio | No |
| ASV_81 | *Vibrio anguillarum* | Vibrio | Vibrio | Vibrio | Vibrio | Vibrio | Vibrio | Yes |
| ASV_82 | *Salinivibrio proteolyticus* | SaliniVibrio | Vibrio | Vibrionaceae | Unclassified | Unclassified | Thaumasiovibrio | No |
| ASV_83 | *Vibrio hispanicus/areninigrae* | Vibrio | Vibrio | Vibrio | Vibrio | Vibrio | Vibrio | Yes |
| ASV_84 | *Vibrio caribbeanicus* | Vibrio | Vibrio | Vibrio | Vibrio | Vibrio | Vibrio | Yes |
| ASV_85 | *Salinivibrio proteolyticus* | SaliniVibrio | SaliniVibrio | Vibrionaceae | SaliniVibrio | Unclassified | SaliniVibrio | Yes |
| ASV_86 | *Grimontia marina* | Grimontia | Grimontia | Grimontia | Vibrionaceae | Enterovibrio | Grimontia | Yes |
| ASV_87 | *Thaumasiovibrio subtropicus* | ThaumasioVibrio | Candidatus Photodesmus | Unclassified | Unclassified | Unclassified | Vibrionaceae | No |
| ASV_88 | *Photobacterium panuliri* | Photobacterium | Photobacterium | Photobacterium | Photobacterium | Photobacterium | Photobacterium | Yes |
| ASV_89 | *Vibrio ippocampi* | Vibrio | Vibrio | Vibrio | Vibrionaceae | Vibrio | Vibrio | Yes |
| ASV_90 | *Vibrio anguillarum* | Vibrio | ThaumasioVibrio | ThaumasioVibrio | Vibrionaceae | Unclassified | Vibrionaceae | No |
| ASV_91 | *Grimontia sp.* | Grimontia | Grimontia | Grimontia | Grimontia | Vibrionaceae | Grimontia | No |
| ASV_92 | *Photobacterium lutimaris* | Photobacterium | Photobacterium | Photobacterium | Photobacterium | Photobacterium | Photobacterium | Yes |
| ASV_93 | *Vibrio cholerae/mimicus* | Vibrio | Vibrio | Vibrio | Vibrio | Vibrio | Vibrio | Yes |
| ASV_94 | *Vibrio chagasii* | Vibrio | Vibrio | Vibrio | Vibrio | Vibrio | Vibrio | Yes |
| ASV_95 | *Vibrio caribbeanicus* | Vibrio | Vibrio | Vibrio | Vibrio | Vibrio | Vibrio | Yes |
| ASV_96 | *Salinivibrio proteolyticus* | Salinivibrio | Vibrio | Vibrionaceae | Unclassified | Unclassified | Thaumasiovibrio | No |
| ASV_97 | *Grimontia sp.* | Grimontia | Vibrionaceae | Grimontia | Grimontia | Grimontia | Vibrionaceae | No |
| ASV_98 | *Vibrio navarrensis* | Vibrio | Vibrio | Vibrio | Vibrionaceae | Vibrio | Vibrio | Yes |
| ASV_99 | *Vibrio anguillarum* | Vibrio | Vibrio | Vibrio | Vibrio | Vibrio | Vibrio | Yes |
| ASV_100 | *Vibrio anguillarum* | Vibrio | Vibrio | Vibrio | Vibrio | Vibrio | Vibrio | Yes |
| ASV_101 | *Aliivibrio fischeri* | Aliivibrio | Aliivibrio | Aliivibrio | Aliivibrio | Aliivibrio | Aliivibrio | Yes |
| ASV_102 | *Vibrio xiamenensis* | Vibrio | Vibrio | Vibrio | Vibrio | Vibrio | Vibrio | Yes |
| ASV_103 | *Vibrio xiamenensis* | Vibrio | Vibrio | Vibrio | Vibrionaceae | Vibrio | Vibrio | Yes |
| ASV_104 | *Photobacterium swingsii/sanguinicancri* | Photobacterium | Photobacterium | Photobacterium | Photobacterium | Photobacterium | Photobacterium | Yes |
| ASV_105 | *Enterovibrio pacificus* | Enterovibrio | Vibrionaceae | Vibrionaceae | Vibrionaceae | Photobacterium | Vibrionaceae | No |
| ASV_106 | *Paraphotobacterium marinum* | Paraphotobacterium | Photobacterium | Photobacterium | Vibrionaceae | Vibrionaceae | Photobacterium | No |
| ASV_107 | *Photobacterium aestuarii* | Photobacterium | Photobacterium | Photobacterium | Photobacterium | Photobacterium | Photobacterium | Yes |
| ASV_108 | *Grimontia marina* | Grimontia | Grimontia | Grimontia | Vibrionaceae | Grimontia | Grimontia | Yes |
| ASV_109 | *Thaumasiovibrio subtropicus* | ThaumasioVibrio | Vibrionaceae | Vibrionaceae | Vibrionaceae | ThaumasioVibrio | Thaumasiovibrio | Yes |
| ASV_110 | *Enterovibrio pacificus* | Enterovibrio | Vibrionaceae | Vibrionaceae | Vibrionaceae | Photobacterium | Vibrionaceae | No |
| ASV_111 | *Enterovibrio coralii* | Enterovibrio | Enterovibrio | Vibrionaceae | Vibrionaceae | Enterovibrio | Enterovibrio | Yes |
| ASV_112 | *Vibrio cholerae* | Vibrio | Vibrio | Vibrio | Vibrio | Vibrio | Vibrio | Yes |
| ASV_113 | *Photobacterium damselae* | Photobacterium | Vibrionaceae | Vibrio | Unclassified | Photobacterium | Photobacterium | Yes |
| ASV_114 | *Enterovibrio pacificus* | Enterovibrio | Photobacterium | Vibrionaceae | Vibrionaceae | Vibrionaceae | Vibrionaceae | No |
| ASV_115 | *Paraphotobacterium marinum* | Paraphotobacterium | Vibrionaceae | Vibrionaceae | Vibrionaceae | Photobacterium | Vibrionaceae | No |
| ASV_116 | *Paraphotobacterium marinum* | Paraphotobacterium | Photobacterium | Photobacterium | Photobacterium | Photobacterium | Photobacterium | No |
| ASV_117 | *Paraphotobacterium marinum* | Paraphotobacterium | Vibrionaceae | Vibrionaceae | Vibrionaceae | Photobacterium | Vibrionaceae | No |
| ASV_118 | *Enterovibrio coralii* | Enterovibrio | Enterovibrio | Enterovibrio | Enterovibrio | Enterovibrio | Enterovibrio | Yes |
| ASV_119 | *Grimontia marina* | Grimontia | Grimontia | Grimontia | Vibrionaceae | Grimontia | Grimontia | Yes |
| ASV_120 | *Photobacterium sp.* | Photobacterium | Photobacterium | Photobacterium | Photobacterium | Photobacterium | Photobacterium | No |
| ASV_121 | *Photobacterium aphoticum* | Photobacterium | Photobacterium | Photobacterium | Photobacterium | Photobacterium | Photobacterium | Yes |
| ASV_122 | *Vibrio pectenicida* | Vibrio | Photobacterium | Photobacterium | Photobacterium | Photobacterium | Photobacterium | No |
| ASV_123 | *Photobacterium sanguinicancri* | Photobacterium | Vibrio | Vibrio | Vibrio | Vibrio | Vibrio | No |
| ASV_124 | *Vibrio ippocampi* | Vibrio | Enterovibrio | Vibrionaceae | Vibrionaceae | Enterovibrio | Vibrionaceae | No |
| ASV_125 | *Enterovibrio pacificus* | Enterovibrio | Vibrio | Vibrionaceae | Gammaproteobacteria | Vibrio | Vibrio | No |
| ASV_126 | *Vibrio anguillarum* | Vibrio | Vibrionaceae | Vibrio | Vibrio | Vibrio | Vibrio | Yes |
| ASV_127 | *Enterovibrio pacificus* | Enterovibrio | Grimontia | Vibrionaceae | Vibrionaceae | Enterovibrio | Enterovibrio | Yes |
| ASV_128 | *Enterovibrio pacificus* | Enterovibrio | Candidatus Photodesmus | Unclassified | Unclassified | Unclassified | Thaumasiovibrio | No |
| ASV_129 | *Enterovibrio coralii* | Enterovibrio | Grimontia | Vibrionaceae | Vibrionaceae | Enterovibrio | Enterovibrio | Yes |
| ASV_130 | *Vibrio chagasii* | Vibrio | Enterovibrio | Enterovibrio | Enterovibrio | Enterovibrio | Enterovibrio | No |
| ASV_131 | *Vibrio chagasii* | Vibrio | Vibrio | Vibrio | Vibrio | Vibrio | Vibrio | Yes |
| ASV_132 | *Photobacterium marinum* | Photobacterium | Photobacterium | Vibrionaceae | Vibrionaceae | Photobacterium | Photobacterium | Yes |

**Table S4.** Taxa in the initial (total) and final database following the phylogenetic placement and *consensus-based* approach

|  | **Species** | **Total** | **Exluded** | **Final** | **% Retained** |
| --- | --- | --- | --- | --- | --- |
| ASV_51, 101 | *Aliivibrio fischeri* | 0.067937175 | 0 | 0.067937 | 100 |
| ASV_41 | *Aliivibrio sp.* | 0.083217491 | 0.083217 | 0 | 0 |
| ASV_16, 19,24,26,34,67 | *Candidatus Photodesmus* | 1.045848948 | 1.045849 | 0 | 0 |
| ASV_42 | *Enterovibrio calviensis* | 0.080482406 | 0 | 0.080482 | 100 |
| ASV_76,111,118,129 | *Enterovibrio coralii* | 0.031503916 | 0 | 0.031504 | 100 |
| ASV_09 | *Enterovibrio norvegicus* | 0.638693832 | 0 | 0.638694 | 100 |
| ASV_105,110,114,125,127,128 | *Enterovibrio pacificus* | 0.016695964 | 0.014859 | 0.001837 | 11 |
| ASV_86,108,119 | *Grimontia marina* | 0.021394442 | 0 | 0.021394 | 100 |
| ASV_91,97 | *Grimontia sp.* | 0.018934758 | 0.018935 | 0 | 0 |
| ASV_66,78,106,115,116,117 | *Paraphotobacterium marinum* | 0.072110192 | 0 | 0.07211 | 100 |
| ASV_107 | *Photobacterium aestuarii* | 0.003813091 | 0 | 0.003813 | 100 |
| ASV_07,12 | *Photobacterium angustum* | 1.610613071 | 0 | 1.610613071 | 100 |
| ASV_22,37,60,62,121 | *Photobacterium aphoticum* | 2.00751109 | 0.308709 | 1.698802 | 85 |
| ASV_13,21,113 | *Photobacterium damselae* | 0.738724752 | 0.227995 | 0.510729 | 69 |
| ASV_52 | *Photobacterium gaetbulicola* | 0.062191735 | 0 | 0.062192 | 100 |
| ASV_92 | *Photobacterium lutimaris* | 0.011439272 | 0 | 0.011439 | 100 |
| ASV_132 | *Photobacterium marinum* | 0.0947409 | 0 | 0.0947409 | 100 |
| ASV_88 | *Photobacterium panuliri* | 0.014280924 | 0 | 0.014281 | 100 |
| ASV_64,74 | *Photobacterium rosenbergii* | 0.064257912 | 0 | 0.064258 | 100 |
| ASV_32 | *Photobacterium sanctipauli* | 0.122687621 | 0 | 0.122688 | 100 |
| ASV_27,123 | *Photobacterium sanguinicancri* | 0.161969673 | 0.002301 | 0.159669 | 99 |
| ASV_14,38,120 | *Photobacterium sp.* | 0.500070352 | 0.50007 | 0 | 0 |
| ASV_15 | *Photobacterium swingsii* | 0.397482141 | 0 | 0.397482 | 100 |
| ASV_104 | *Photobacterium swingsii/sanguinicancri* | 0.004079441 | 0 | 0.004079 | 100 |
| ASV_30 | *Photobacterium_atrarenae* | 0.126084548 | 0 | 0.126085 | 100 |
| ASV_43,82,85,96 | *Salinivibrio proteolyticus* | 0.120534774 | 0.105437 | 0.015097 | 13 |
| ASV_55,87,109 | *Thaumasiovibrio subtropicus* | 0.074702068 | 0.014707 | 0.059995 | 80 |
| ASV_33,56,71,75,77,81,90,99,100,126,44 | *Vibrio anguillarum* | 0.287713789 | 0.01211 | 0.275604 | 96 |
| ASV_44 | *Vibrio anguillarum/ordalii* | 0.075779203 | 0 | 0.075779 | 100 |
| ASV_57 | *Vibrio atypicus* | 0.048196039 | 0 | 0.048196 | 100 |
| ASV_08,11,17,20,84,95 | *Vibrio caribbeanicus* | 1.763172226 | 0 | 1.763172 | 100 |
| ASV_01,23,29,40,70,94,130,131 | *Vibrio chagasii* | 26.35108508 | 0.001526 | 26.34956 | 100 |
| ASV_112 | *Vibrio cholerae* | 0.003513549 | 0 | 0.003514 | 100 |
| ASV_93 | *Vibrio cholerae/mimicus* | 0.009685699 | 0 | 0.009686 | 100 |
| ASV_65 | *Vibrio diazotrophicus* | 0.0374518 | 0 | 0.037452 | 100 |
| ASV_25 | *Vibrio estuarianus* | 0.197899059 | 0 | 0.197899 | 100 |
| ASV_31,53 | *Vibrio fluvialis* | 0.185242129 | 0 | 0.185242 | 100 |
| ASV_49,83 | *Vibrio hispanicus/areninigrae* | 0.083862904 | 0 | 0.083863 | 100 |
| ASV_59 | *Vibrio inusitatus* | 0.045536073 | 0 | 0.045536 | 100 |
| ASV_28,48,89,124 | *Vibrio ippocampi* | 0.228063263 | 0.002132 | 0.225932 | 99 |
| ASV_06 | *Vibrio kanaloae/splendidus/lentus* | 1.758167358 | 0 | 1.758167 | 100 |
| ASV_63,98 | *Vibrio navarrensis* | 0.046327959 | 0 | 0.046328 | 100 |
| ASV_10 | *Vibrio orientalis* | 0.63025447 | 0 | 0.630254 | 100 |
| ASV_04 | *Vibrio owensii* | 3.88804532 | 0 | 3.888045 | 100 |
| ASV_73,122,05,47 | *Vibrio pectenicida/tapetis* | 1.943063857 | 0.002436 | 1.940628 | 100 |
| ASV_46 | *Vibrio ponticus* | 0.072840975 | 0 | 0.072841 | 100 |
| ASV_02,03,35,39,45,50,54,61,68,79,80 | *Vibrio sp.* | 13.30183735 | 13.30184 | 0 | 0 |
| ASV_72 | *Vibrio sp. (cholerae clade)* | 0.026487108 | 0 | 0.026487 | 100 |
| ASV_69 | *Vibrio sp. (diazotrophicus clade)* | 0.031129381 | 0 | 0.031129 | 100 |
| ASV_18,36 | *Vibrio splendidus* | 0.353506905 | 0 | 0.353507 | 100 |
| ASV_102,103 | *Vibrio xiamenensis* | 0.004142045 | 0 | 0.004142 | 100 |

**Table S5.** *Consensus-based* abundance (average ± SD) of Vibrionaceae species identified in the different sites. S: Surface, B: Bottom; LS: Lignano Sabbiadoro, SG: San Giorgio di Nogaro, SW: seawater, DP: depurator

| Taxa | **C1_S** | **C1_B** | **LS_SW** | **LS_WW** | **SG_DP** | **SG_DP** |
| --- | --- | --- | --- | --- | --- | --- |
| *Aliivibrio fischeri* | 0.0003±0.00174 | 0.00125±0.00436 | 0.00059±0.00206 | 0 | 0.00069±0.00241 | 0 |
| *Enterovibrio calviensis* | 0 | 0.00005±0.0003 | 0.00276±0.00838 | 0 | 0.0038±0.0111 | 0 |
| *Enterovibrio coralii* | 0 | 0 | 0.00099±0.00232 | 0 | 0.00164±0.00283 | 0 |
| *Enterovibrio norvegicus* | 0.00102±0.00352 | 0.00065±0.00273 | 0.02471±0.07524 | 0 | 0.02381±0.06956 | 0 |
| *Enterovibrio pacificus* | 0 | 0 | 0.00008±0.00027 | 0 | 0.00007±0.00026 | 0 |
| *Grimontia marina* | 0 | 0.00044±0.00195 | 0.00032±0.0011 | 0 | 0.00022±0.00077 | 0 |
| *Photobacterium aestuarii* | 0 | 0 | 0.00032±0.0011 | 0 | 0 | 0 |
| *Photobacterium angustum* | 0.02107±0.08328 | 0.02539±0.08446 | 0.00154±0.00202 | 0 | 0.00103±0.00194 | 0 |
| *Photobacterium aphoticum* | 0.00148±0.00644 | 0.00111±0.00648 | 0 | 0 | 0 | 0 |
| *Photobacterium atrarenae* | 0.00027±0.00159 | 0 | 0.00494±0.01149 | 0 | 0.0048±0.00956 | 0 |
| *Photobacterium damselae* | 0.00943±0.04987 | 0.0054±0.02617 | 0.00028±0.00096 | 0 | 0.00026±0.00061 | 0 |
| *Photobacterium gaetbulicola* | 0.00046±0.00213 | 0.00007±0.00042 | 0.00147±0.00343 | 0 | 0.00221±0.00251 | 0 |
| *Photobacterium lutimaris* | 0 | 0 | 0.00095±0.0033 | 0 | 0 | 0 |
| *Photobacterium marinum* | 0 | 0 | 0 | 0 | 0.00008±0.00027 | 0 |
| *Photobacterium panuliri* | 0 | 0.00042±0.00245 | 0 | 0 | 0 | 0 |
| *Photobacterium rosenbergii* | 0.00097±0.00507 | 0.00014±0.00085 | 0.00129±0.00277 | 0 | 0.0009±0.00185 | 0 |
| *Photobacterium sanctipauli* | 0.00136±0.00595 | 0.00161±0.00641 | 0.00075±0.00148 | 0 | 0.00108±0.00225 | 0 |
| *Photobacterium sanguinicancri* | 0 | 0.00029±0.00167 | 0.00667±0.02312 | 0 | 0.00582±0.02016 | 0 |
| *Photobacterium swingsii* | 0.00027±0.00159 | 0.00004±0.00022 | 0.00114±0.00317 | 0.03066±0.0512 | 0.00033±0.00081 | 0.00011±0.00039 |
| *Photobacterium swingsii/sanguinicancri* | 0 | 0 | 0.00027±0.00063 | 0 | 0.00007±0.00026 | 0 |
| *Salinivibrio proteolyticus* | 0.0001±0.00055 | 0 | 0.00099±0.00342 | 0 | 0 | 0 |
| *Thaumasiovibrio subtropicus* | 0.00049±0.00288 | 0.00011±0.00065 | 0.00224±0.00619 | 0 | 0.00105±0.003 | 0 |
| *Vibrio anguillarum* | 0.00055±0.00237 | 0.00096±0.00371 | 0.01236±0.03899 | 0.00206±0.00495 | 0.00428±0.0101 | 0 |
| *Vibrio anguillarum/ordalii* | 0 | 0.00021±0.0012 | 0.00286±0.00857 | 0 | 0.00287±0.00569 | 0 |
| *Vibrio atypicus* | 0.00023±0.00135 | 0.00112±0.00655 | 0.00018±0.00061 | 0 | 0 | 0 |
| *Vibrio caribbeanicus* | 0.00819±0.0194 | 0.01334±0.03458 | 0.05225±0.16083 | 0.00012±0.0004 | 0.03346±0.09153 | 0.00007±0.00026 |
| *Vibrio chagasii* | 0.18124±0.47159 | 0.37652±1.50805 | 0.35495±1.1056 | 0.0018±0.0027 | 0.25695±0.83689 | 0.00178±0.00276 |
| *Vibrio cholerae* | 0 | 0 | 0 | 0.00029±0.00101 | 0 | 0 |
| *Vibrio cholerae/mimicus* | 0 | 0 | 0 | 0.00081±0.0028 | 0 | 0 |
| *Vibrio diazotrophicus* | 0 | 0 | 0 | 0.00312±0.00567 | 0 | 0 |
| *Vibrio estuarianus* | 0.00202±0.00715 | 0.00287±0.01381 | 0.00201±0.00697 | 0 | 0.00061±0.00212 | 0 |
| *Vibrio fluvialis* | 0 | 0.00345±0.02014 | 0.00287±0.00808 | 0 | 0.00278±0.00962 | 0 |
| *Vibrio hispanicus* | 0.00155±0.00902 | 0.00025±0.00108 | 0 | 0 | 0.00031±0.00107 | 0 |
| *Vibrio hispanicus/areninigrae* | 0 | 0 | 0.00057±0.00136 | 0 | 0.00093±0.00199 | 0.00008±0.00027 |
| *Vibrio inusitatus* | 0.00098±0.00446 | 0.00009±0.00052 | 0.00078±0.00269 | 0 | 0 | 0 |
| *Vibrio ippocampi* | 0.00039±0.00229 | 0 | 0.01049±0.02766 | 0 | 0.00722±0.01944 | 0 |
| *Vibrio kanaloae/splendidus/lentus* | 0.00929±0.01829 | 0.03987±0.11264 | 0.003±0.0104 | 0 | 0.0042±0.01456 | 0 |
| *Vibrio navarrensis* | 0 | 0 | 0 | 0.00386±0.00962 | 0 | 0 |
| *Vibrio orientalis* | 0.00014±0.00079 | 0.00116±0.0056 | 0.0255±0.03199 | 0.00033±0.00064 | 0.02294±0.02612 | 0.00008±0.00026 |
| *Vibrio owensii* | 0.07036±0.20377 | 0.04372±0.18394 | 0.00034±0.00117 | 0 | 0.00046±0.0011 | 0 |
| *Vibrio pectenicida* | 0.0003±0.00172 | 0.00014±0.00055 | 0.00045±0.00094 | 0 | 0.00051±0.00178 | 0 |
| *Vibrio pectenicida/tapetis* | 0.00086±0.00404 | 0.02101±0.11256 | 0.06394±0.0681 | 0.00027±0.00064 | 0.03311±0.03433 | 0.00024±0.00059 |
| *Vibrio ponticus* | 0.0011±0.00535 | 0.00104±0.00606 | 0 | 0 | 0 | 0 |
| *Vibrio sp. (cholerae clade)* | 0 | 0 | 0 | 0.00221±0.00411 | 0 | 0 |
| *Vibrio sp. (diazotrophicus clade)* | 0 | 0 | 0.00177±0.00276 | 0 | 0.00082±0.00138 | 0 |
| *Vibrio splendidus* | 0.00355±0.02068 | 0.00475±0.0228 | 0.00495±0.01079 | 0 | 0.00102±0.00225 | 0 |
| *Vibrio xiamenensis* | 0.00012±0.00071 | 0.00013±0.00077 |  | 0 | 0 | 0 |
